# Do what matters, no matter what! Factorizing positive activities during COVID-19 lockdown

**DOI:** 10.1177/13591053221120967

**Published:** 2022-09-20

**Authors:** Philipp Yorck Herzberg, Tanja Stender, Janina Charlotte Gabriela Dechmann, Jasmin Čolić, Jürgen Hoyer

**Affiliations:** 1 Helmut-Schmidt-Universität/Universität der Bundeswehr Hamburg, Germany; 2 Technische Universität Dresden, Germany

**Keywords:** behavioral activation, mental health, ordinal factor analysis, positive activities, reflective vs formative measurement

## Abstract

Behavioral activation (BA) interventions systematically encourage positive and value-based activities. Engaging in them is an effective way to counteract negative affect, but it is unknown whether there are subtypes of activities that may have differential effects on mood. This study investigated the factorial structure of 99 potentially rewarding activities used in an online BA intervention during the COVID-19 lockdown. About 3624 German-speaking participants evaluated a list of 99 activities that were easy to apply. We analyzed the initially 99 activities by means of confirmatory factor analysis. Since activities can either be seen as reflective or formative indicators, a reflective as well as a formative model was analyzed. Although the range of chosen activities differed clearly between respondents, a one-factor model provided the best fit. It seems that a general “activity” factor is more important for explaining whether people choose a certain activity or not, than specific characteristics of the activity itself.

The coronavirus disease 2019 (COVID-19) pandemic has direct and indirect health, social, and economic effects that are assumed to also negatively impact on mental health. As a central characteristic of the crisis, regulations intending to minimize social contacts, thus, the risk of infection, more or less restricted the range of daily activities. From a psychological perspective, this bares a tremendous risk for the individual to lose opportunities for positive reward. The regulations during the COVID-19 lockdown imply, in other words, a lack of reinforcement as they dramatically restrict the range of possible activities. However, even if you are forced to stay at home and to avoid close face-to-face social contacts, numerous alternative activities remain that are potentially rewarding.

It is unclear, however, which kinds of activities are to be preferred when an effect on mood and well-being is intended. For instance, while both physical exercise (e.g. Chan et al., 2019; Reed and Ones, 2006) and mindfulness-oriented activities (e.g. Howarth et al., 2019; Rodrigues et al., 2017) have consistently been shown to improve mood, differential effects of the two are noticeable. Anaerobic exercise mainly proved to augment energetic arousal (Kanning and Schlicht, 2010) and reduce inertia/fatigue, while mindfulness more broadly affected negative emotions (going beyond just mood; Edwards and Loprinzi, 2018; Müller et al., 2021). The combination of both (i.e. mindful exercise) seemed to be most effective (Chan et al., 2019). Furthermore, activities that are self-determined or intrinsically motivated may have more pronounced effects than those having to do with obligations (or forms of extrinsic motivation; Laran and Janiszewski, 2011; Meyer et al., 2021). If activities are social in nature or not, may also impact mood, with social activities being beneficial across a variety of clinical and non-clinical conditions (e.g. Fingerman et al., 2020; Holt-Lunstad et al., 2010; Kamarsu et al., 2020; Paolillo et al., 2018). Considerations like these imply that activities leading to reward experiences are heterogeneous and might be determined by a broad range of diverse psychological factors (e.g. Rider et al., 2016, for a sample of Alzheimer’s Disease patients see Amspoker et al., 2019). Looking at the lists of rewarding activities that have been developed since the 1970s in the context of behavioral activation interventions (see below), all of the above mentioned potential prototypes of rewarding behaviors are included. However, at least to our knowledge, the factorial structure underlying activities during the COVID-19 lockdown has until now never been empirically explored.

More specifically, there is scant literature about what kind of activities people chose and performed during the COVID-19 lockdown (e.g. Harikrishnan and Sailo, 2020; Harikrishnan et al., 2020). Results show a preference of using social networking sites (30.7%), reading books (15.2%), and cooking (13.2%) in a sample of young Indians. There are also indications of a preference for housework (24.5%) and exercise (19.2%) in the group of physical activities and a preference for group/video chats (20.3%), looking at old photos (14.3%) in the group of social activities. However, sedentary behaviors increased significantly whereas physical activities decreased significantly during the COVID-19 lockdown in a sample of 1430 students in Italy (Gallè et al., 2020). Likewise, in a large study in Switzerland (*N* = 5932), aged 18–75+ years, a substantial share of participants reported to feel depressed (33%) and anxious (43%) more often during the COVID-19 lockdown than before, and 45% of the participants reported a decreased frequency of physical activity during the lockdown compared to before (Hansmann et al., 2021). Wickersham et al. (2021) reported changes in physical activity among United Kingdom university students. The largest data base data (*N* = 11,775) according physical activity among adults during the COVID-19 lockdown was collected by Ding et al. (2021). They found substantial reductions in physical activity levels during COVID-19 lockdown across 11 countries and concluded that physical activity promotion interventions are needed during this and similar global emergencies.

In a preceding study (Hoyer et al., 2021), we were interested in how much and which activities people choose when reminded of a certain set of activities. We presented a list of potentially rewarding activities online, giving respondents the chance to select from this list and to broaden the range of positive and/or rewarding activities that could enhance reinforcement rates even during a typical COVID-19 lockdown day. We referred to the Pleasant Events Schedule (PES; MacPhillamy and Lewinsohn, 1982) as a template for a list of positive activities. This list, developed in the 1970s, is very long (containing 320 items) and had to be carefully revised and shortened (to no more than 99 items of activities that can be done even under lockdown restrictions, most prominently at home; see below).

The factorial structure of either the PES or newer versions of such activity lists is, at least to our knowledge, unknown. The question of the factorial structure of potentially rewarding activities is, however, interesting and relevant with reference to the planning of BA interventions, regardless whether informal and self-applied or formal and expert-applied. Based on the data set mentioned above, we will therefore apply advanced factor analytical methods to address the question of the dimensional structure of rewarding activities during COVID-19 lockdown. There are two reasons to presume a multi-dimensional structure of the rewarding activities list. First, given the large number of 99 activities, it is reasonable to assume a multifactorial structure which an unknown number of latent factors. Second, comparable lists of actions, responses, or other behaviors are predominantly multidimensional (see Wilkinson, 2013).

## Methods

### Study design and sample

The web-based study was cross-sectional with an integrated intervention component. It was conducted with SoSci Survey (Leiner, 2019). Recruitment and data collection occurred between April 9th, 2020 and April 26th, 2020. The participants were recruited through social media, online and newspaper articles. All individuals had to be at least 16 years old and had to have sufficient knowledge of the German language to participate in the study. To encourage the recruitment, all participants had the opportunity to download the list with 99 rewarding activities at the end of the survey. The participation was anonymous, voluntary and non-commercial. All participants gave their consent on the first survey page, and the study was conducted in accordance with the declaration of Helsinki. Based on the fact that data were completely anonymous and the IP-addresses of the respondents were not stored, the ethics committee of the Technische Universität Dresden deemed further formal evaluation of the study not necessary.

A total of *N* = 3625 German-speaking subjects participated. One participant was excluded subsequently because of unserious answers. The final sample consisted of *N* = 3624 participants. A subsample of *N* = 2561 (71%) engaged in an imagined BA exercise (IBA, see below). Table 1 shows the sociodemographic and clinical characteristics of the sample.

**Table 1.** Sociodemographic and clinical characteristics of the sample (*N* = 3624).

Characteristics	Descriptives
Age, M (SD)	36.84 (11.75)
Sex, %	
Female	83.2
Male	16.3
Diverse	0.5
Years of education, %	
<8	0.4
8–10	18.4
11–13	29.2
14+	51.9
Employment status, %	
Still in school/university	18.2
Full-time employed	39.3
Part-time employed	21.2
Unemployed	3.0
Retired	2.9
On parental leave/other exemptions	7.1
Living arrangements, %	
Living alone	19.8
Shared apartment	8.7
Living with spouse	29.7
Living with family	41.7
Number of children, %	
0	60.55
1	16.91
2	17.16
3+	5.38
More time at home in %, M (SD)	58.28 (29.13)
Change of work situation, %	
Loss of employment	4.4
Reduced working hours	9.4
Increased working hours	5.0
Change to home-office	48.4
No change at all	32.7
Double burden (work and childcare), %	21.9
Risk of severe course of COVID-19, %	20
Personally infected by COVID-19, %	0.4
Flat mate infected by COVID-19, %	0.3
PANAS pre, M (SD)	
PA	2.61 (0.69)
NA	1.89 (0.67)
PHQ-9, M (SD)	7.58 (5.31)
GAD-7, M (SD)	6.26 (4.79)
PANAS post, M (SD)	
PA	2.69 (0.84)
NA	1.44 (0.61)

COVID-19: Coronavirus disease 2019; PANAS: Positive and Negative Affect Schedule; PA: positive affect; NA: negative affect; PHQ-9: Patient Health Questionnaire-9; GAD-7 = Generalized Anxiety Disorder-7.

### Measures

Participants completed questions about demographics, COVID-19 related burdens, affect, depression, and anxiety. Supplemental Table X1 (Supplemental Materials) shows the full variable list in. The German versions of the instruments were used.

#### Activity list

Hoyer and Krämer (2021) reduced and refreshed the PES (MacPhillamy and Lewinsohn, 1982) with 320 positive activities to 301 items. Since many activities were not viable due to the COVID-19 related restrictions in everyday life, four independent raters indicated on a scale from 0 to 2 how suitable each activity from the list was (0 = *unsuitable*, 1 = *only suitable to a limited extent*, 2 = *suitable*). One-hundred thirty-seven items reached the criterion of a sum score of seven points (across all four raters). We sorted out 21 items because of unspecific, unsuitable or too specific content (e.g. “having leisure time,” “using virtual reality glasses”). Because of doublings and similarities, we reduced 45 items to 11 items by combining their content (e.g. “listening to an audiobook” and “listening to a podcast” combined to “listening to an audiobook or podcast”). We also modified 35 existing items (e.g. “going shopping” to “online shopping”) to suit the COVID-19 related restrictions. We discussed 23 additional self-provided COVID-19 suitable items and included 17 of them (e.g. “streaming an online lecture,” “watching a movie in a foreign language”). In the end, the list was shortened and supplemented to 99 implementable activities (that can be done even under lockdown restrictions, most prominently at home). For the complete list, see the electronic Supplemental Material (Table 2).

**Table 2.** Activity list and frequencies.

	Activities	Relative frequency (%)	CFA λ
1.	Watch a movie/a tv series	70,05	0.399
2.	Do household chores	66,62	-
3.	Go for a walk	66,62	0.413
4.	Clean and tidy up thoroughly	64,53	0.424
5.	Talk/chat with friends/family over phone/skype	64,53	0.497
6.	Do sports (e.g. a home workout/stretching)	60,91	0.473
7.	Shower/take a bath	59,42	0.414
8.	Bake	55,61	0.470
9.	Try a new cooking recipe	51,29	0.517
10	Chat over social media	48,90	0.474
11	Listen to an audio book/podcast	48,04	0.505
12	Finish a task or a project	48,00	0.442
13	Ride a bicycle	45,77	0.490
14	Do gardening	45,32	0.473
15	Create a vegetable patch or repot a plant	43,53	0.475
16	Make plans for the post-lockdown period	41,18	0.583
17	Put clean sheets on the bed	40,06	-
18	Enjoy an extensive breakfast/brunch	39,76	0.480
19	Jog or do outdoor exercises	39,43	0.486
20	Look at or sort old photos	39,31	0.593
21	Read novels, short stories, plays or poems	38,57	0.494
22	Sort out or upgrade clothing	38,42	0.337
23	Do meditation or yoga	38,38	0.433
24	Brighten up the apartment or renovate a room	37,67	0.506
25	Research on the Internet on a subject	36,59	0.493
26	Groom myself	36,59	0.584
27	Act artistically (painting, sculpturing, drawing)	36,26	0.491
28	Shop online	35,43	0.492
29	Read magazines or newspapers	35,40	0.599
30.	Play board or card games	34,43	0.484
31.	Listen to music consciously (not only incidentally)	33,98	0.573
32.	Organize or archive something	32,53	-
33.	Knit, crochet, embroider or sew	32,41	-
34.	Implement a long-delayed task (e.g. tax return)	32,26	0.567
35.	Playing puzzles, solving crosswords, etc.	31,93	0.501
36.	Take time for a long conversation	31,56	0.663
37.	Read a textbook or nonfiction	31,41	0.669
38.	Look at stars, moon or clouds	31,22	0.501
39.	Prepare/make a lovely gift	31,22	0.496
40.	Watch funny videos, listen to jokes or read jokes	29,28	-
41.	Compliment or praise someone	29,06	0.504
42.	Enjoy sexuality in partnership	28,57	0.515
43.	Walk barefoot	27,79	0.542
44.	Make my parents happy	27,75	0.598
45.	Dedicate myself intensively to the children	27,42	0.683
46.	Play/learn a musical instrument	26,74	0.614
47.	Play games on smartphone	26,22	0.576
48.	Watch the sunrise or sunset	25,77	0.613
49.	Sing	25,03	0.484
50.	Write letters or cards	24,88	0.561
51.	Take care of my financial affairs	24,62	0.515
52.	Play video games (e.g. computer, Playstation, Wii)	24,58	0.487
53.	Play with pets	24,51	0.569
54.	Learn/practice a foreign language	23,80	0.633
55.	Dance for myself	23,42	-
56.	Think of an encouragement for others	22,79	0.607
57.	Enjoy sexuality alone	22,60	0.523
58.	Do a mindfulness exercise	22,01	0.632
59.	Create a healthy diet plan	21,52	0.541
60.	Donate to a good cause	21,11	0.467
61.	Plan breaks actively	20,48	-
62.	Address a personal problem	20,44	0.619
63.	Sell something online	20,25	0.621
64.	Preserve, freeze and stockpile food	19,96	0.573
65.	Work on technical things (car, bicycle, motorcycle, household appliances)	18,87	0.548
66.	Watching a film in a foreign language	18,76	0.388
67.	Work with artistic materials (clay, leather, pearls, wool, etc.)	18,58	0.543
68.	Make a photo album	18,50	0.388
69.	Photograph	18,35	0.403
70.	Put on make-up, fix my hair, etc.	18,05	0.430
71.	Offer an advice or help	17,49	0.384
72.	Watch and imitate do-it-yourself videos	17,20	0.591
73.	Write a diary	17,12	0.484
74.	Read tips and advice for self-help	15,33	0.403
75.	Stream an online presentation	15,03	0.568
76.	Restore antiques, refurbish furniture	14,81	-
77.	Watch a video of a concert	14,47	0.633
78.	Listen to the radio attentively	13,91	0.392
79.	Post photos in social media	13,73	0.521
80.	Learn something new (e.g. juggling, football tricks, handstand)	13,20	0.531
81.	Study for an exam	12,27	0.591
82.	Organize a party with old friends via internet	11,97	0.593
83.	Start a new hobby	10,74	0.591
84.	Apply for a new job	10,59	0.627
85.	Write stories, plays or poems	9,03	0.472
86.	Ask someone for advice or help	8,06	0.592
87.	Pick out TV shows thoroughly	7,87	0.513
88.	Study maps/learn capitals	7,09	0.576
89.	Write a blog article or post a video	5,30	0.551
90.	Shoot or edit a video	5,22	0.553
91.	Maintain my homepage	4,70	0.580
92.	Medically fast	4,51	0.474
93.	Compose a song or a piece of music	4,40	0.557
94.	Sing karaoke (by yourself or with friends/family)	3,43	0.576
95.	Learn a magic trick	3,06	0.383
96.	Participate in internet corona challenges (e.g. #oldphotochallenge)	2,91	0.436
97.	Confess something to somebody	1,79	0.427
98.	Conduct a fashion show at home	1,64	0.541
99.	Write/recite a poem	1,16	0.490

λ: Factor loadings from the second sample. – indicates, that the item was removed from the final list.

#### Positive and negative affect

We measured the subjects’ state affect with the Positive and Negative Affect Schedule (PANAS; Watson et al., 1988; German version: Krohne et al., 1996). It consists of two 10-item-scales to measure positive affect (PA; e.g. active, enthusiastic) and negative affect (NA; e.g. afraid, distressed). Participants were asked how they felt in the exact moment using a Likert scale ranging from 1 (=*not at all*) to 5 (=*very much*). A total score for PA and NA (mean across all PA items and all NA items) can be derived. The German version of the PANAS showed high reliability (Cronbach’s α = 0.86–0.93) for positive and negative affect (Breyer and Bluemke, 2016). Positive and negative affect (PANAS) were assessed before and after completing a list of rewarding activities and engaging in the IBA.

#### Depression

Symptoms of depression were measured with the Patient Health Questionnaire-9 (PHQ-9; Kroenke and Spitzer, 2002; German version: Löwe et al., 2004). It is based on the Diagnostic and Statistical Manual of Mental Disorders (DSM-IV; APA, 1994) and consists of nine items depicting diagnostic criteria of depression over the past 2 weeks (e.g. a loss of interest, feelings of depression or hopelessness). The participants could indicate their answers on a 4-point Likert scale ranging from 0 (=*not at all*) to 3 (=*nearly every day*). The total score ranges from 0 to 27, with higher scores indicating more severity of depressive symptoms (Kroenke and Spitzer, 2002). The German version of the PHQ-9 showed high reliability (Cronbach’s α = 0.88; Gräfe et al., 2004).

#### Anxiety

Symptoms of anxiety were measured with the Generalized Anxiety Disorder-7 (GAD-7; Spitzer et al., 2006; German version: Löwe et al., 2008). It is based on the DSM-IV (APA, 1994) and consists of seven items depicting diagnostic criteria of generalized anxiety disorder over the past 2 weeks (e.g. feelings of anxiety or nervousness, worrying about different things). The participants could indicate their answers on a 4-point Likert scale ranging from 0 (=*not at all*) to 3 (=*nearly every day*). The score ranges from 0 to 21 with higher scores indicating a more severe course of anxiety symptoms (Löwe et al., 2008). The German version of the GAD-7 showed high reliability (Cronbach’s α = 0.89; Löwe et al., 2008). The PHQ-9 and the GAD-7 are two modules of the Patient Health Questionnaire (Spitzer, 1999).

### Statistical analysis

Before conducting the statistical analysis of the activity list, we had to decide whether the activities are assumed to be formative or reflective indicators of the latent construct. Reflective constructs have observed measures that are affected by the underlying latent construct. Formative constructs are a composite of multiple measures. Unlike reflective measures, where a change in the construct affects the underlying measures, changes in the formative measures cause changes in the underlying construct (see Diamantopoulos and Siguaw, 2006). According to Edwards (2011) we decided to adopt a two-step approach. First, we analyzed the initial item pool of the 99 activities by means of exploratory factor analysis (EFA), in order to gain first insights into the factor structure of the activity list. Next, the resulting model will be cross-validated by means of a confirmatory factor analysis (CFA). Both approaches treating the activity items as reflective indicators. This allows us to apply the sophisticated toolbox of covariance-based scale development (Coaley, 2014; Noar, 2003) to optimize the scale independently from a given criterion that is needed in formative scale construction. The rationale is that formative constructs in isolation are statistically under-identified due to “indeterminacies associated with the scale of measurement for the latent construct and the construct level error term” (Jarvis et al., 2003: 213). To achieve identification, a formative construct must be placed within a model which contains structural relationships. However, the structural relationships may affect the measurement model. To minimize the impact of the structural model on the measurement model, we used reflective measurement approach to obtain “pure” psychometric parameters of the scale. However, to address the issue that reflective specification for a set of formative indicators of an endogenous construct may lead to underestimation of the structural parameters for its effects. Therefore, we compared the structural relationships of the activity scale with anxiety and depression yielded by the accompanied structural equation approach (SEM) for the reflective model and the accompanied partial least squares approach (PLS) for the formative model. Reflective models were analyzed with Mplus and the formative model with SmartPLS 3 (Ringle et al., 2015).

Several fit-indices were examined to evaluate the overall fit of each model. First, the chi-square goodness-of-fit statistic was reported. However, the chi-square statistic is sensitive to sample size, so it is rarely used as a sole index of model fit. Therefore, three incremental indices of fit were examined: the normed fit index (NFI), the comparative fit index (CFI), and the Tucker-Lewis index (TLI). Incremental indices reflected the improvement in fit gained by a given factor model relative to the most restrictive (null or independence) model. All three incremental indices are scaled from 0 (no fit) to 1 (perfect fit). Hu and Bentler (1999) advised that values close to 0.95 are indicative of a good fit. Finally, the root mean square error of approximation (RMSEA) is a population discrepancy function that compensates for the effects of model complexity. The closer the RMSEA coefficient is to 0, the better the fit of the model. According to Browne and Cudeck (1993), a RMSEA value of 0.05 or less indicates a close fit of the model in relation to the degrees of freedom, whereas a value of 0.08 or less indicates a reasonable error of approximation. Since the variables were binary and the sample size was sufficiently large, analyses were based on diagonally weighted least squares estimations (WLSMV) with robust standard errors. This approach takes the categorical nature of the variables into account, and also considers that categorical variables violate the assumption of multivariate normality in CFA. Furthermore, it ensures unbiased parameter estimations and standard errors (Beauducel and Herzberg, 2006).

Given the large sample size, a CFA was conducted on half of the sample (*n* = 1812) and a further confirmatory factor analysis was conducted on the second half (*n* = 1812) based on the results of the first sample. The second sample was used for cross-validation of the activity list by a second CFA and to investigate the relationship with anxiety and depression. We assumed a negative relationship between activity and anxiety and depression, respectively.

## Results

### Exploratory factor analyses

Although the Kaiser-Meyer-Olkin criterion (KMO) of 0.909 and Bartlett’s test of sphericity with χ^2^(4851) = 23,186.3, *p* < 0.001 indicate the appropriateness of the 99 activities for an EFA, the number of factors suggested by the different factor retention criteria varied between 1 (Hull method and lower bound of RMSEA 90% confidence interval) and 18 (parallel analysis with 2000 replications), the MAP Test suggested 6 factors. None of the multi-dimensional solutions resulted in an interpretable factor solution (low factor loadings, several secondary factor loadings of equal sizes etc.) with factors that pass the reliability requirement of at least 0.70 per scale. Given that two criteria suggested a one-factor solution (Hull method and lower bound of RMSEA 90% confidence interval), we next tested whether the activity list covers a single dimension. Therefore, we estimated a one-factor model with an CFA to assess uni-dimensionality and to delete items with low factor loadings (<0.30).

### Confirmatory factor analyses

The one-factor model yielded a statistically significant chi-square goodness-of-fit statistic (χ^2^ = 12,191.40, df = 4752, *p* < 0.001), suggesting a poor fit to the data. However, the chi-square statistic is sensitive to sample size, so it is rarely used as a sole index of model fit (Hu and Bentler, 1999). In terms of the absolute model fit indices, the one-factor model provided a good fit to the data, RMSEA = 0.029, 90% CI of RMSEA 0.029−0.030, SRMR = 0.085. However, in terms of relative model fit, indices provided a reasonable fit to the data, CFI = 0.852, TLI = 0.849. Factor loadings ranged from 0.12 to 0.69. Eight items with loadings <0.30 were excluded (Item 2, 17, 32, 33, 40, 55, 61, 76).

This reduced item pool was tested on the other half of the sample (*N* = 1812). The modified model met the evaluation criteria for a good fit with regard to all fit-statistics considered, except for the chi-square goodness-of-fit statistic (χ^2^ = 10385.99, df = 4004, *p* < 0.001). In terms of the absolute model fit indices, the modified one-factor model provided a good fit to the data with RMSEA = 0.030, 90% CI of RMSEA 0.029−0.030, SRMR = 0.080. The relative model fit was good with CFI = 0.943, TLI = 0.941. Factor loadings ranged from 0.34 to 0.68. Factor loadings are given in Table 2.

### Descriptive statistics

The final activity list contains 91 activities. Table 3 provides an overview of the descriptive analyses of the activity list. In addition to the mean we reported the percent of maximal possible values, which are easier to interpret than the mean. On the average, the participants of our study reported that the conducted about 64% of the mentioned activities with a standard deviation of about 8%. A significant Kolmogorov-Smirnov-test (0.095, *p* < 0.001) revealed that the list is not normally distributed. Internal consistency was assessed with Cronbach’s alpha, McDonald’s omega and split half reliability. Cronbach’s alpha and McDonald’s omega are calculated from polychoric correlations. Cronbach’s alpha was 0.97, McDonald’s omega was 0.94 (95% CI with 1000 bootstrap replications = 0.93−0.94) and split half was 0.95. As an alternative estimate of reliability we computed Guttman bounds reliability which was 0.96.

**Table 3.** Descriptive Analyses of the activity list.

	Estimate
Mean	1.271
SD	0.16
POMP	63.69
SD	7.91
Median	62.09
Modus	59.89
Minimum	50.00
Maximum	97.25
Skewness	0.94
Kurtosis	0.82

POMP: percent of maximal possible.

### Activity and its latent correlations

In a first attempt to infer about the validity of the activity list, and concomitantly the impact of the selected activities, we tested whether higher self-reported activity levels are related to lower self-rated anxiety and depression levels. All analyses were done as latent variable analyses. First, we conducted the analysis as a covariance analysis in a SEM-framework, treating all variables as reflective measures with ordinal indicators (estimator was WLSMV with robust standard errors). Model fit for the SEM model was good with χ^2^ = 12,563.06, df = 5667, *p* < 0.001, RMSEA = 0.026,90% CI of RMSEA 0.025−0.027, SRMR = 0.077, CFI = 0.892, and TLI = 0.890. Latent path coefficients were −0.09 (*p* < 0.001) for depression and −0.10 (*p* < 0.001) for anxiety, respectively. The latent correlation between depression and anxiety was 0.87 (*p* < 0.001).

Second, we repeated the analysis in a PLS-framework, treating the activity list as a formative measure and depression and anxiety as reflective measures. Model fit for the PLS model was good with χ^2^ = 3368.59, df = 5667, *p* < 0.001, SRMR = 0.014, NFI = 0.935. All Variance Inflation Factors (VIF) were <2. Composite reliability for depression was 0.85 and 0.88 for anxiety, respectively. The latent correlation between depression and anxiety was 0.88 (*p* < .001). Latent path coefficients between activity and depression was −0.43 (*p* < 0.001) for and −0.02 (*p* > 0.05) for anxiety, respectively. The bootstrapped path coefficients with 2000 replications were −0.47 (95% CI −0.43 to −0.41) between activity and depression and −0.04 (95% CI −0.06 to 0.03) between activity and anxiety, respectively. Please note that the coefficients are larger than in the SEM-analyses, which is due to the prediction-oriented focus of the PLS approach compared to the theory testing or theory confirmation focus of the SEM-framework (Rigdon et al., 2017).

Finally, we tested whether reporting activities would change affect. We assume that reporting activities enhances positive affect and diminish negative affect. Given the well-known limitations of simple difference scores (e.g. Cronbach and Furby, 1970) we used latent difference scores modeling (LDS) to test the impact of conducting rewarding activities on affect. We used a multiple indicator bivariate dual latent change score model (Ghisletta and Lindenberger, 2003) to test the assumed increase in positive affect and decrease in negative effect through the conduct of rewarding activities. As above, all variables were handled as ordinal indicators (estimator was WLSMV with robust standard errors). Model fit for the SEM model was good with χ^2^ = 28,551.40, df = 8310, *p* < 0.001, RMSEA = 0.037,90% CI of RMSEA 0.037−0.038, SRMR = 0.073, CFI = 0.960, and TLI = 0.959. Latent path coefficients were 0.20 (*p* < 0.001) on the latent difference score for positive affect and −0.11 (*p* < 0.01) on the latent difference score for negative affect, respectively. The latent correlation between both difference scores was −0.23 (*p* < 0.001). Results indicated that a small but significant increase in positive affect is associated with exercise of rewarding activities as well as a corresponding decrease in negative affect.

## Discussion

The factorial structure of a list with 99 activities that could be chosen and performed during a COVID-19 lockdown was explained by a single factor. There is no indication of statistically robust sub-factors of activity. This was true despite the fact that the items we factorized included a wide range of diverging activities, from physical exercise to mindfulness-oriented activities and from those augmenting energetic arousal to those inducing relaxation. Also, intrinsically motivated versus extrinsically motivated activities and social versus private activities could be chosen from the list. Nevertheless, these a priori categories did not seem to be statistically distinguishable, contradicting previous findings by Wilkinson (2013). Hence, psychologically the most important difference between activities seems to be, whether they lead to some form of reward (“do what matters, no matter what!”) or not. As neuroimaging studies have shown, there is empirical evidence that any positive experience and any kind of reward is converted into a “common currency” of pleasure (Berridge and Kringelbach, 2015). The present data seems to confirm this idea on a different level of observation, namely self-report.

Although we can only refer to cross-sectional, correlational data, small to moderate associations between activity and negative and positive affect as well as more trait like depression could be demonstrated. There was no subset of items (factor) that would be more closely associated with the abovementioned outcomes than others. This implies that in constrained circumstances, such as a lockdown, it is not important for our well-being *what* specific activities we undertake, but rather *that* we engage in any meaningful activities at all. The individuals affected nevertheless have differential preferences for activities (“do what matters (to you). . .”). However, as the “common currency” hypothesis suggests, all these activities may produce the same pattern of pleasure in the brain (“. . .no matter what!”; Berridge and Kringelbach, 2015). Given that understanding, “activities” do not need to directly induce pleasure or involve physical activity. Actions that are mood stabilizing would include duties and daily chores as well as “doing nothing,” that is, relaxing and regenerative activities. All of these can have the same motivating and mood-enhancing effect, as long as individuals are free to choose between them. This has important implications, not only for a lockdown situation but also for the treatment of mood disorders.

In brief, a lack of positive reward is one of the most important anteceding and maintaining factors of depression (Hoyer et al., 2021; Lejuez et al., 2011; Lewinsohn, 1974). To counteract this deficit, the method of choice is behavioral activation (BA; Hoyer et al., 2020a, 2020b; Martell et al., 2013). BA aims to increase the amount of rewarding activities in the daily lives of depressed patients through activity monitoring and activity planning (Hoyer and Krämer, 2021). The present results suggest that any kind of activity might be sufficient to produce the desired effects, at least in the beginning phases of the intervention. However, further research with clinical populations is needed to confirm these assumptions and their clinical relevance.

Several limitations need to be outlined and discussed. First, the list of activities was specific to those applicable during a lockdown (most prominently at home). To this aim, we reduced the PES and adapted it to COVID-19 related restrictions in Germany (Hoyer et al., 2021). In addition, to increase feasibility and reduce dropout rates in the online study, we limited the list to only 99 activities. Future studies should include a larger and more comprehensive list of activities, which includes those relevant for communities in other parts of the world. Secondly, the sample is not representative of the German population, as it was predominantly female, college-educated and included those interested in partaking in online-studies. Future studies should aim to gather a more stratified sample.

Despite its shortcomings, the present study had several strengths. One of the main advantages is the large sample. The study included more than 3600 subjects from the general population with a wide age range and differential mental health status. Namely, 20%–30% of the sample were at least mildly impaired by depressive and/or generalized anxiety symptoms. This allows for broader conclusions that go beyond those from previous studies investigating the factorial structure of the PES, which included highly selective samples of elderly people or those with Alzheimer’s disease (Amspoker et al., 2019; Rider et al., 2016). Another strength is that the list proved to be easy to understand and apply, given that thousands of respondents used it without issues.

In sum, we developed and factor-analyzed a list of activities that proved useful in promoting healthy and meaningful activities even in a restricted setting, such as the COVID-19 lockdown. We showed that executing activities from this list helps to reduce negative affect, self-rated anxiety and depression and, to a lesser degree, enhance positive affect. Interestingly, no subtype of activity was identified that would specifically serve these functions; rather activity itself seems to be what is important. This list can be used by a wide variety of instructors, like coaches, psychotherapists, or geriatric nurses, in a wide variety of settings (e.g. internet- and mobile based interventions or face-to-face counseling) and it may prove its usefulness in the event that other pandemics arise.

## Supplemental Material

sj-docx-1-hpq-10.1177_13591053221120967 – Supplemental material for Do what matters, no matter what! Factorizing positive activities during COVID-19 lockdown Click here for additional data file. Supplemental material, sj-docx-1-hpq-10.1177_13591053221120967 for Do what matters, no matter what! Factorizing positive activities during COVID-19 lockdown by Philipp Yorck Herzberg, Tanja Stender, Janina Charlotte Gabriela Dechmann, Jasmin Čolić and Jürgen Hoyer in Journal of Health Psychology

sj-docx-2-hpq-10.1177_13591053221120967 – Supplemental material for Do what matters, no matter what! Factorizing positive activities during COVID-19 lockdown Click here for additional data file. Supplemental material, sj-docx-2-hpq-10.1177_13591053221120967 for Do what matters, no matter what! Factorizing positive activities during COVID-19 lockdown by Philipp Yorck Herzberg, Tanja Stender, Janina Charlotte Gabriela Dechmann, Jasmin Čolić and Jürgen Hoyer in Journal of Health Psychology

sj-pdf-3-hpq-10.1177_13591053221120967 – Supplemental material for Do what matters, no matter what! Factorizing positive activities during COVID-19 lockdown Click here for additional data file. Supplemental material, sj-pdf-3-hpq-10.1177_13591053221120967 for Do what matters, no matter what! Factorizing positive activities during COVID-19 lockdown by Philipp Yorck Herzberg, Tanja Stender, Janina Charlotte Gabriela Dechmann, Jasmin Čolić and Jürgen Hoyer in Journal of Health Psychology

sj-png-4-hpq-10.1177_13591053221120967 – Supplemental material for Do what matters, no matter what! Factorizing positive activities during COVID-19 lockdown Click here for additional data file. Supplemental material, sj-png-4-hpq-10.1177_13591053221120967 for Do what matters, no matter what! Factorizing positive activities during COVID-19 lockdown by Philipp Yorck Herzberg, Tanja Stender, Janina Charlotte Gabriela Dechmann, Jasmin Čolić and Jürgen Hoyer in Journal of Health Psychology

sj-sav-5-hpq-10.1177_13591053221120967 – Supplemental material for Do what matters, no matter what! Factorizing positive activities during COVID-19 lockdown Click here for additional data file. Supplemental material, sj-sav-5-hpq-10.1177_13591053221120967 for Do what matters, no matter what! Factorizing positive activities during COVID-19 lockdown by Philipp Yorck Herzberg, Tanja Stender, Janina Charlotte Gabriela Dechmann, Jasmin Čolić and Jürgen Hoyer in Journal of Health Psychology

sj-sav-6-hpq-10.1177_13591053221120967 – Supplemental material for Do what matters, no matter what! Factorizing positive activities during COVID-19 lockdown Click here for additional data file. Supplemental material, sj-sav-6-hpq-10.1177_13591053221120967 for Do what matters, no matter what! Factorizing positive activities during COVID-19 lockdown by Philipp Yorck Herzberg, Tanja Stender, Janina Charlotte Gabriela Dechmann, Jasmin Čolić and Jürgen Hoyer in Journal of Health Psychology This article is distributed under the terms of the Creative
Commons Attribution 4.0 License ( http://www.creativecommons.org/licenses/by/4.0/) which
permits any use, reproduction and distribution of the work without
further permission provided the original work is attributed as specified
on the SAGE and Open Access pages ( https://us.sagepub.com/en-us/nam/open-access-at-sage).
