# Supplementary material for: Do what matters, no matter what! Factorizing positive activities during COVID-19 lockdown
Source: J Health Psychol. 2022 Sep 20;28(5):477–90. doi: 10.1177/13591053221120967 (PMC9490392; doi:10.1177/13591053221120967)
Supplement: sj-docx-1-hpq-10.1177_13591053221120967 – Supplemental material for Do what matters, no matter what! Factorizing positive activities during COVID-19 lockdown [file sj-docx-1-hpq-10.1177_13591053221120967.docx]

**Supplemental material**

Table X1

*Description of the survey questions and response options*

| **Survey questions** | **Response options** |
| --- | --- |
| **Sociodemographic variables** |  |
| Age |  |
| Sex | Female |
|  | Male |
|  | Diverse |
| Years of education |  |
| < 8 | Without graduation |
| 8-10 | Certificate of compulsory secondary education |
|  | General certificate of secondary education |
| 11-13 | Subject-related entrance qualification |
|  | Higher education entrance qualification (A-levels) |
| 14+ | Bachelor’s degree |
|  | Master’s degree |
|  | PhD |
| Employment status | Still in school/university |
|  | Full-time employed |
|  | Part-time employed |
|  | Unemployed |
|  | Retired |
|  | On parental leave/ other exemptions |
| Location | State in Germany |
|  | Abroad |
| Living arrangement | Living alone |
|  | Shared apartment |
|  | Living with spouse |
|  | Living with family |
| Number of children |  |
| **COVID-19 related burdens** |  |
| More time at home | in % |
| Change of work situation | Loss of employment |
|  | Reduced working hours |
|  | Increased working hours |
|  | Change to home-office |
|  | No change at all |
| Double burden (work and childcare) | Yes |
|  | No |
| Risk of severe course of COVID-19 | Yes |
|  | No |
| Personally infected by COVID-19 | Yes |
|  | No |
| Flat mate infected by COVID-19 | Yes |
|  | No |
| **PA and NA pre** | PANAS, 20 items, scale 1-5 |
| **Depression** | PHQ-9; 9 items, DSM-IV criteria for MDD, scale 0-3 |
| **Anxiety** | GAD-7; 7 items, DSM-IV criteria for GAD, scale 0-3 |
| **Activity List** | Activity list with 99 items |
| Selected rewarding activities | Yes |
|  | No |
| Came up with new ideas for activities | Yes, one from the list |
|  | Yes, other |
|  | No |
| Motivation | Scale 0-10 |
| **IBA** | Audio |
| Listened to audio | Yes |
|  | No |
| Chosen voice | Female |
|  | Male |
| Which activity |  |
| Quality of implementation | Scale 0-10 |
| Increase of implementation | Yes |
|  | No |
| **PA and NA post** | PANAS, 20 items, scale 1-5 |
| *Note*. COVID-19 = Coronavirus disease 2019; PA = positive affect; NA = negative affect; PANAS = Positive and Negative Affect Schedule; PHQ-9 = Patient Health Questionnaire-9; DSM-IV = Diagnostic and Statistical Manual of Mental Disorders; MDD = major depressive disorder; GAD-7 = Generalized Anxiety Disorder-7; GAD = generalized anxiety disorder; IBA = imaginary behavioral activation. | |
