# Supplementary material for: Do what matters, no matter what! Factorizing positive activities during COVID-19 lockdown
Source: J Health Psychol. 2022 Sep 20;28(5):477–90. doi: 10.1177/13591053221120967 (PMC9490392; doi:10.1177/13591053221120967)
Supplement: sj-docx-2-hpq-10.1177_13591053221120967 – Supplemental material for Do what matters, no matter what! Factorizing positive activities during COVID-19 lockdown [file sj-docx-2-hpq-10.1177_13591053221120967.docx]

**Subsample 0:**

Reflektiv:

modelref <- " Activity =~ ak06_01 + ak06_02 + ak06_03 + ak06_04 + ak06_05 + ak06_06 + ak06_07 + ak06_08 + ak06_09 + ak06_10 + ak06_11 + ak06_12 + ak06_13 + ak06_14 + ak06_15 + ak06_16 + ak06_17 + ak06_18 + ak06_19 + ak06_20 + ak06_21 + ak06_22 + ak06_23 + ak06_24 + ak06_25 + ak06_26 + ak06_27 + ak06_28 + ak06_29 + ak06_30 + ak06_31 + ak06_32 + ak06_33 + ak06_34 + ak06_35 + ak06_36 + ak06_37 + ak06_38 + ak06_39 + ak06_40 + ak06_41 + ak06_42 + ak06_43 + ak06_44 + ak06_45 + ak06_46 + ak06_47 + ak06_48 + ak06_49 + ak06_50 + ak06_51 + ak06_52 + ak06_53 + ak06_54 + ak06_55 + ak06_56 + ak06_57 + ak06_58 + ak06_59 + ak06_60 + ak06_61 + ak06_62 + ak06_63 + ak06_64 + ak06_65 + ak06_66 + ak06_67 + ak06_68 + ak06_69 + ak06_70 + ak06_71 + ak06_72 + ak06_73 + ak06_74 + ak06_75 + ak06_76 + ak06_77 + ak06_78 + ak06_79 + ak06_80 + ak06_81 + ak06_82 + ak06_83 + ak06_84 + ak06_85 + ak06_86 + ak06_87 + ak06_88 + ak06_89 + ak06_90 + ak06_91 + ak06_92 + ak06_93 + ak06_94 + ak06_95 + ak06_96 + ak06_97 + ak06_98 + ak06_99

Depression =~ de02_01 + de02_02 + de02_03 + de02_04 + de02_05 + de02_06 + de02_07 + de02_08 + de02_09

Anxiety =~ an01_01 + an01_02 + an01_03 + an01_04 + an01_05 + an01_06 + an01_07

Depression ~~ Anxiety

Activity ~ Depression + Anxiety

"

fit <- sem(model = modelref, data = data, ordered = TRUE)

summary(fit, standardized = TRUE, fit.measures = TRUE)

semPaths(fit, "std", title = FALSE)

lavaanPlot(model = fit, labels = labels, node_options = list(shape = "box", fontname = "Helvetica"), edge_options = list(color = "grey"), coefs = TRUE)

Estimator DWLS

Optimization method NLMINB

Number of model parameters 265

Number of observations 1812

Model Test User Model:

Standard Robust

Test Statistic 24058.791 14520.334

Degrees of freedom 6437 6437

P-value (Chi-square) 0.000 0.000

Scaling correction factor 2.328

Shift parameter 4183.610

simple second-order correction

Model Test Baseline Model:

Test statistic 273159.624 77702.723

Degrees of freedom 6555 6555

P-value 0.000 0.000

Scaling correction factor 3.747

User Model versus Baseline Model:

Comparative Fit Index (CFI) 0.934 0.886

Tucker-Lewis Index (TLI) 0.933 0.884

Robust Comparative Fit Index (CFI) NA

Robust Tucker-Lewis Index (TLI) NA

Root Mean Square Error of Approximation:

RMSEA 0.039 0.026

90 Percent confidence interval - lower 0.038 0.026

90 Percent confidence interval - upper 0.039 0.027

P-value RMSEA <= 0.05 1.000 1.000

Robust RMSEA NA

90 Percent confidence interval - lower NA

90 Percent confidence interval - upper NA

Standardized Root Mean Square Residual:

SRMR 0.082 0.082

Parameter Estimates:

Standard errors Robust.sem

Information Expected

Information saturated (h1) model Unstructured

Latent Variables:

Estimate Std.Err z-value P(>|z|) Std.lv Std.all

Activity =~

ak06_01 1.000 0.442 0.442

ak06_02 0.338 0.082 4.140 0.000 0.150 0.150

ak06_03 0.954 0.077 12.461 0.000 0.422 0.422

ak06_04 0.694 0.089 7.759 0.000 0.307 0.307

ak06_05 1.003 0.084 11.933 0.000 0.443 0.443

ak06_06 0.868 0.103 8.411 0.000 0.384 0.384

ak06_07 1.106 0.085 13.044 0.000 0.489 0.489

ak06_08 1.131 0.088 12.803 0.000 0.500 0.500

ak06_09 1.201 0.086 14.032 0.000 0.531 0.531

ak06_10 1.105 0.081 13.569 0.000 0.488 0.488

ak06_11 1.077 0.094 11.516 0.000 0.476 0.476

ak06_12 0.981 0.088 11.092 0.000 0.434 0.434

ak06_13 1.023 0.087 11.819 0.000 0.452 0.452

ak06_14 1.177 0.088 13.358 0.000 0.520 0.520

ak06_15 1.004 0.082 12.266 0.000 0.444 0.444

ak06_16 1.409 0.098 14.390 0.000 0.623 0.623

ak06_17 0.655 0.130 5.022 0.000 0.290 0.290

ak06_18 1.063 0.096 11.118 0.000 0.470 0.470

ak06_19 1.187 0.083 14.301 0.000 0.525 0.525

ak06_20 1.225 0.089 13.733 0.000 0.541 0.541

ak06_21 1.092 0.152 7.179 0.000 0.483 0.483

ak06_22 0.877 0.083 10.589 0.000 0.388 0.388

ak06_23 1.048 0.083 12.642 0.000 0.463 0.463

ak06_24 1.127 0.097 11.665 0.000 0.498 0.498

ak06_25 1.070 0.092 11.646 0.000 0.473 0.473

ak06_26 1.411 0.095 14.871 0.000 0.624 0.624

ak06_27 1.126 0.091 12.376 0.000 0.498 0.498

ak06_28 1.195 0.088 13.597 0.000 0.528 0.528

ak06_29 1.402 0.092 15.166 0.000 0.620 0.620

ak06_30 1.131 0.088 12.788 0.000 0.500 0.500

ak06_31 1.245 0.095 13.064 0.000 0.550 0.550

ak06_32 0.629 0.080 7.884 0.000 0.278 0.278

ak06_33 0.463 0.076 6.115 0.000 0.205 0.205

ak06_34 1.139 0.086 13.242 0.000 0.503 0.503

ak06_35 1.164 0.092 12.686 0.000 0.515 0.515

ak06_36 1.447 0.095 15.256 0.000 0.640 0.640

ak06_37 1.484 0.095 15.593 0.000 0.656 0.656

ak06_38 1.093 0.090 12.077 0.000 0.483 0.483

ak06_39 1.116 0.179 6.220 0.000 0.493 0.493

ak06_40 0.639 0.075 8.491 0.000 0.282 0.282

ak06_41 1.166 0.083 14.098 0.000 0.515 0.515

ak06_42 1.191 0.090 13.286 0.000 0.526 0.526

ak06_43 1.298 0.088 14.782 0.000 0.574 0.574

ak06_44 1.424 0.094 15.158 0.000 0.630 0.630

ak06_45 1.546 0.101 15.367 0.000 0.683 0.683

ak06_46 1.395 0.105 13.224 0.000 0.616 0.616

ak06_47 1.143 0.088 13.011 0.000 0.505 0.505

ak06_48 1.361 0.096 14.128 0.000 0.601 0.601

ak06_49 1.000 0.083 12.060 0.000 0.442 0.442

ak06_50 1.266 0.092 13.782 0.000 0.560 0.560

ak06_51 1.295 0.117 11.095 0.000 0.573 0.573

ak06_52 1.082 0.098 11.076 0.000 0.478 0.478

ak06_53 1.170 0.090 12.936 0.000 0.517 0.517

ak06_54 1.448 0.099 14.596 0.000 0.640 0.640

ak06_55 0.167 0.074 2.270 0.023 0.074 0.074

ak06_56 1.378 0.091 15.115 0.000 0.609 0.609

ak06_57 1.176 0.098 12.022 0.000 0.520 0.520

ak06_58 1.273 0.092 13.899 0.000 0.563 0.563

ak06_59 1.186 0.094 12.570 0.000 0.524 0.524

ak06_60 1.009 0.083 12.186 0.000 0.446 0.446

ak06_61 0.507 0.074 6.815 0.000 0.224 0.224

ak06_62 1.350 0.099 13.584 0.000 0.597 0.597

ak06_63 1.363 0.096 14.182 0.000 0.602 0.602

ak06_64 1.282 0.090 14.171 0.000 0.567 0.567

ak06_65 1.094 0.101 10.827 0.000 0.483 0.483

ak06_66 0.994 0.093 10.734 0.000 0.440 0.440

ak06_67 1.283 0.092 13.966 0.000 0.567 0.567

ak06_68 0.844 0.094 9.018 0.000 0.373 0.373

ak06_69 0.965 0.085 11.314 0.000 0.427 0.427

ak06_70 1.025 0.088 11.634 0.000 0.453 0.453

ak06_71 0.886 0.081 10.922 0.000 0.392 0.392

ak06_72 1.431 0.113 12.692 0.000 0.633 0.633

ak06_73 1.196 0.089 13.493 0.000 0.529 0.529

ak06_74 0.803 0.119 6.772 0.000 0.355 0.355

ak06_75 1.161 0.088 13.178 0.000 0.513 0.513

ak06_76 0.813 0.099 8.179 0.000 0.359 0.359

ak06_77 1.390 0.108 12.881 0.000 0.614 0.614

ak06_78 0.960 0.110 8.705 0.000 0.424 0.424

ak06_79 1.300 0.092 14.194 0.000 0.574 0.574

ak06_80 1.182 0.091 12.937 0.000 0.522 0.522

ak06_81 1.254 0.091 13.801 0.000 0.554 0.554

ak06_82 1.203 0.101 11.965 0.000 0.532 0.532

ak06_83 1.312 0.092 14.254 0.000 0.580 0.580

ak06_84 1.138 0.194 5.875 0.000 0.503 0.503

ak06_85 0.685 0.152 4.510 0.000 0.303 0.303

ak06_86 1.343 0.087 15.458 0.000 0.594 0.594

ak06_87 1.267 0.098 12.918 0.000 0.560 0.560

ak06_88 1.393 0.193 7.207 0.000 0.616 0.616

ak06_89 1.135 0.096 11.838 0.000 0.502 0.502

ak06_90 1.227 0.096 12.838 0.000 0.542 0.542

ak06_91 1.388 0.088 15.692 0.000 0.614 0.614

ak06_92 0.953 0.086 11.118 0.000 0.421 0.421

ak06_93 1.022 0.117 8.775 0.000 0.452 0.452

ak06_94 1.545 0.138 11.194 0.000 0.683 0.683

ak06_95 0.808 0.120 6.706 0.000 0.357 0.357

ak06_96 0.843 0.079 10.660 0.000 0.372 0.372

ak06_97 0.847 0.078 10.844 0.000 0.374 0.374

ak06_98 1.227 0.090 13.572 0.000 0.543 0.543

ak06_99 0.871 0.122 7.143 0.000 0.385 0.385

Depression =~

de02_01 1.000 0.723 0.723

de02_02 1.198 0.026 46.464 0.000 0.865 0.865

de02_03 0.871 0.028 30.996 0.000 0.630 0.630

de02_04 1.045 0.026 40.044 0.000 0.755 0.755

de02_05 0.820 0.030 27.590 0.000 0.593 0.593

de02_06 1.036 0.029 36.244 0.000 0.749 0.749

de02_07 0.931 0.027 34.610 0.000 0.673 0.673

de02_08 0.880 0.035 25.231 0.000 0.636 0.636

de02_09 1.053 0.037 28.527 0.000 0.761 0.761

Anxiety =~

an01_01 1.000 0.799 0.799

an01_02 1.136 0.018 64.804 0.000 0.907 0.907

an01_03 1.105 0.017 64.804 0.000 0.882 0.882

an01_04 1.050 0.018 59.489 0.000 0.839 0.839

an01_05 0.773 0.025 30.875 0.000 0.617 0.617

an01_06 0.843 0.022 39.193 0.000 0.673 0.673

an01_07 0.944 0.018 51.735 0.000 0.754 0.754

Regressions:

Estimate Std.Err z-value P(>|z|) Std.lv Std.all

Activity ~

Depression -0.095 0.049 -1.953 0.051 -0.156 -0.156

Anxiety 0.026 0.043 0.616 0.538 0.048 0.048

Covariances:

Estimate Std.Err z-value P(>|z|) Std.lv Std.all

Depression ~~

Anxiety 0.507 0.015 34.935 0.000 0.879 0.879

**CROSS-Validation:**

Estimator DWLS

Optimization method NLMINB

Number of model parameters 251

Number of observations 1812

Model Test User Model:

Standard Robust

Test Statistic 19358.171 12563.061

Degrees of freedom 5667 5667

P-value (Chi-square) 0.000 0.000

Scaling correction factor 2.154

Shift parameter 3576.198

simple second-order correction

Model Test Baseline Model:

Test statistic 265608.423 69613.393

Degrees of freedom 5778 5778

P-value 0.000 0.000

Scaling correction factor 4.070

User Model versus Baseline Model:

Comparative Fit Index (CFI) 0.947 0.892

Tucker-Lewis Index (TLI) 0.946 0.890

Robust Comparative Fit Index (CFI) NA

Robust Tucker-Lewis Index (TLI) NA

Root Mean Square Error of Approximation:

RMSEA 0.037 0.026

90 Percent confidence interval - lower 0.036 0.025

90 Percent confidence interval - upper 0.037 0.027

P-value RMSEA <= 0.05 1.000 1.000

Robust RMSEA NA

90 Percent confidence interval - lower NA

90 Percent confidence interval - upper NA

Standardized Root Mean Square Residual:

SRMR 0.077 0.077

Parameter Estimates:

Standard errors Robust.sem

Information Expected

Information saturated (h1) model Unstructured

Latent Variables:

Estimate Std.Err z-value P(>|z|) Std.lv Std.all

Activity =~

ak06_01 1.000 0.398 0.398

ak06_03 1.036 0.085 12.162 0.000 0.413 0.413

ak06_04 1.060 0.115 9.249 0.000 0.423 0.423

ak06_05 1.247 0.101 12.401 0.000 0.497 0.497

ak06_06 1.179 0.121 9.757 0.000 0.470 0.470

ak06_07 1.048 0.089 11.801 0.000 0.417 0.417

ak06_08 1.173 0.099 11.899 0.000 0.467 0.467

ak06_09 1.290 0.104 12.442 0.000 0.514 0.514

ak06_10 1.185 0.097 12.275 0.000 0.472 0.472

ak06_11 1.248 0.114 10.989 0.000 0.497 0.497

ak06_12 1.102 0.103 10.651 0.000 0.439 0.439

ak06_13 1.231 0.108 11.370 0.000 0.490 0.490

ak06_14 1.191 0.099 12.025 0.000 0.475 0.475

ak06_15 1.201 0.097 12.324 0.000 0.478 0.478

ak06_16 1.464 0.113 12.976 0.000 0.583 0.583

ak06_18 1.186 0.109 10.840 0.000 0.473 0.473

ak06_19 1.234 0.096 12.822 0.000 0.491 0.491

ak06_20 1.483 0.109 13.563 0.000 0.591 0.591

ak06_21 1.229 0.169 7.264 0.000 0.490 0.490

ak06_22 0.843 0.090 9.410 0.000 0.336 0.336

ak06_23 1.092 0.097 11.318 0.000 0.435 0.435

ak06_24 1.265 0.109 11.567 0.000 0.504 0.504

ak06_25 1.245 0.106 11.698 0.000 0.496 0.496

ak06_26 1.478 0.107 13.798 0.000 0.589 0.589

ak06_27 1.230 0.110 11.215 0.000 0.490 0.490

ak06_28 1.244 0.103 12.086 0.000 0.496 0.496

ak06_29 1.518 0.107 14.174 0.000 0.605 0.605

ak06_30 1.211 0.099 12.209 0.000 0.483 0.483

ak06_31 1.424 0.112 12.737 0.000 0.567 0.567

ak06_34 1.416 0.104 13.569 0.000 0.564 0.564

ak06_35 1.263 0.108 11.732 0.000 0.503 0.503

ak06_36 1.677 0.117 14.285 0.000 0.668 0.668

ak06_37 1.678 0.118 14.213 0.000 0.669 0.669

ak06_38 1.254 0.111 11.343 0.000 0.500 0.500

ak06_39 1.231 0.207 5.935 0.000 0.491 0.491

ak06_41 1.275 0.098 12.957 0.000 0.508 0.508

ak06_42 1.291 0.100 12.931 0.000 0.514 0.514

ak06_43 1.365 0.102 13.364 0.000 0.544 0.544

ak06_44 1.520 0.110 13.864 0.000 0.605 0.605

ak06_45 1.719 0.122 14.063 0.000 0.685 0.685

ak06_46 1.536 0.128 11.966 0.000 0.612 0.612

ak06_47 1.441 0.109 13.230 0.000 0.574 0.574

ak06_48 1.532 0.117 13.102 0.000 0.610 0.610

ak06_49 1.216 0.108 11.259 0.000 0.485 0.485

ak06_50 1.412 0.108 13.065 0.000 0.563 0.563

ak06_51 1.278 0.136 9.433 0.000 0.509 0.509

ak06_52 1.223 0.116 10.536 0.000 0.487 0.487

ak06_53 1.411 0.112 12.577 0.000 0.562 0.562

ak06_54 1.589 0.119 13.342 0.000 0.633 0.633

ak06_56 1.512 0.112 13.482 0.000 0.602 0.602

ak06_57 1.312 0.114 11.552 0.000 0.523 0.523

ak06_58 1.591 0.116 13.669 0.000 0.634 0.634

ak06_59 1.352 0.113 11.952 0.000 0.539 0.539

ak06_60 1.182 0.097 12.133 0.000 0.471 0.471

ak06_62 1.560 0.121 12.844 0.000 0.621 0.621

ak06_63 1.551 0.115 13.487 0.000 0.618 0.618

ak06_64 1.447 0.106 13.600 0.000 0.577 0.577

ak06_65 1.376 0.125 11.002 0.000 0.548 0.548

ak06_66 0.973 0.112 8.693 0.000 0.388 0.388

ak06_67 1.363 0.110 12.374 0.000 0.543 0.543

ak06_68 0.966 0.118 8.181 0.000 0.385 0.385

ak06_69 1.015 0.095 10.676 0.000 0.405 0.405

ak06_70 1.084 0.108 10.043 0.000 0.432 0.432

ak06_71 0.971 0.093 10.434 0.000 0.387 0.387

ak06_72 1.445 0.135 10.712 0.000 0.576 0.576

ak06_73 1.217 0.100 12.161 0.000 0.485 0.485

ak06_74 1.007 0.147 6.845 0.000 0.401 0.401

ak06_75 1.420 0.110 12.881 0.000 0.566 0.566

ak06_76 0.614 0.110 5.572 0.000 0.245 0.245

ak06_77 1.584 0.129 12.232 0.000 0.631 0.631

ak06_78 0.987 0.121 8.142 0.000 0.393 0.393

ak06_79 1.318 0.100 13.234 0.000 0.525 0.525

ak06_80 1.339 0.110 12.189 0.000 0.533 0.533

ak06_81 1.490 0.111 13.383 0.000 0.594 0.594

ak06_82 1.482 0.122 12.152 0.000 0.591 0.591

ak06_83 1.487 0.111 13.399 0.000 0.593 0.593

ak06_84 1.581 0.227 6.971 0.000 0.630 0.630

ak06_85 1.176 0.173 6.788 0.000 0.469 0.469

ak06_86 1.485 0.105 14.142 0.000 0.592 0.592

ak06_87 1.291 0.116 11.113 0.000 0.514 0.514

ak06_88 1.440 0.224 6.434 0.000 0.574 0.574

ak06_89 1.387 0.118 11.754 0.000 0.553 0.553

ak06_90 1.390 0.118 11.791 0.000 0.554 0.554

ak06_91 1.450 0.104 13.915 0.000 0.578 0.578

ak06_92 1.186 0.104 11.436 0.000 0.473 0.473

ak06_93 1.395 0.149 9.348 0.000 0.556 0.556

ak06_94 1.438 0.169 8.498 0.000 0.573 0.573

ak06_95 0.962 0.150 6.414 0.000 0.383 0.383

ak06_96 1.096 0.090 12.208 0.000 0.437 0.437

ak06_97 1.082 0.094 11.470 0.000 0.431 0.431

ak06_98 1.360 0.107 12.662 0.000 0.542 0.542

ak06_99 1.223 0.140 8.709 0.000 0.487 0.487

Depression =~

de02_01 1.000 0.730 0.730

de02_02 1.200 0.026 45.829 0.000 0.876 0.876

de02_03 0.888 0.027 32.748 0.000 0.648 0.648

de02_04 1.036 0.027 38.967 0.000 0.756 0.756

de02_05 0.773 0.030 25.722 0.000 0.565 0.565

de02_06 1.001 0.030 33.655 0.000 0.731 0.731

de02_07 0.978 0.027 36.428 0.000 0.714 0.714

de02_08 0.815 0.037 22.097 0.000 0.595 0.595

de02_09 0.987 0.040 24.873 0.000 0.721 0.721

Anxiety =~

an01_01 1.000 0.806 0.806

an01_02 1.127 0.016 70.353 0.000 0.909 0.909

an01_03 1.087 0.016 69.994 0.000 0.877 0.877

an01_04 1.012 0.017 60.532 0.000 0.816 0.816

an01_05 0.772 0.025 30.623 0.000 0.622 0.622

an01_06 0.835 0.021 39.768 0.000 0.673 0.673

an01_07 0.894 0.020 45.442 0.000 0.720 0.720

Regressions:

Estimate Std.Err z-value P(>|z|) Std.lv Std.all

Depression ~

Activity -0.174 0.050 -3.483 0.000 -0.095 -0.095

Anxiety ~

Activity -0.200 0.054 -3.672 0.000 -0.099 -0.099

Covariances:

Estimate Std.Err z-value P(>|z|) Std.lv Std.all

.Depression ~~

.Anxiety 0.507 0.015 33.194 0.000 0.870 0.870

**Subsample 1:**

library(haven)

data <- read_sav("C:/Users/herzberg/Me/Artikel/Hoyer/2021/Sub_1.sav")

View(data)

model2 <- " Activity =~ ak06_01 + ak06_03 + ak06_04 + ak06_05 + ak06_06 + ak06_07 + ak06_08 + ak06_09 + ak06_10 + ak06_11 + ak06_12 + ak06_13 + ak06_14 + ak06_15 + ak06_16 + ak06_18 + ak06_19 + ak06_20 + ak06_21 + ak06_22 + ak06_23 + ak06_24 + ak06_25 + ak06_26 + ak06_27 + ak06_28 + ak06_29 + ak06_30 + ak06_31 + ak06_34 + ak06_35 + ak06_36 + ak06_37 + ak06_38 + ak06_39 + ak06_41 + ak06_42 + ak06_43 + ak06_44 + ak06_45 + ak06_46 + ak06_47 + ak06_48 + ak06_49 + ak06_50 + ak06_51 + ak06_52 + ak06_53 + ak06_54 + ak06_56 + ak06_57 + ak06_58 + ak06_59 + ak06_60 + ak06_62 + ak06_63 + ak06_64 + ak06_65 + ak06_66 + ak06_67 + ak06_68 + ak06_69 + ak06_70 + ak06_71 + ak06_72 + ak06_73 + ak06_74 + ak06_75 + ak06_77 + ak06_78 + ak06_79 + ak06_80 + ak06_81 + ak06_82 + ak06_83 + ak06_84 + ak06_85 + ak06_86 + ak06_87 + ak06_88 + ak06_89 + ak06_90 + ak06_91 + ak06_92 + ak06_93 + ak06_94 + ak06_95 + ak06_96 + ak06_97 + ak06_98 + ak06_99 "

fit <- sem(model = model2, data = data, ordered = TRUE)

summary(fit, standardized = TRUE, fit.measures = TRUE)

semPaths(fit, "std", title = FALSE)

.ak06_22 0.000 0.000 0.000

.ak06_23 0.000 0.000 0.000

.ak06_24 0.000 0.000 0.000

.ak06_25 0.000 0.000 0.000

.ak06_26 0.000 0.000 0.000

.ak06_27 0.000 0.000 0.000

.ak06_28 0.000 0.000 0.000

.ak06_29 0.000 0.000 0.000

.ak06_30 0.000 0.000 0.000

.ak06_31 0.000 0.000 0.000

.ak06_34 0.000 0.000 0.000

.ak06_35 0.000 0.000 0.000

.ak06_36 0.000 0.000 0.000

.ak06_37 0.000 0.000 0.000

.ak06_38 0.000 0.000 0.000

.ak06_39 0.000 0.000 0.000

.ak06_41 0.000 0.000 0.000

.ak06_42 0.000 0.000 0.000

.ak06_43 0.000 0.000 0.000

.ak06_44 0.000 0.000 0.000

.ak06_45 0.000 0.000 0.000

.ak06_46 0.000 0.000 0.000

.ak06_47 0.000 0.000 0.000

.ak06_48 0.000 0.000 0.000

.ak06_49 0.000 0.000 0.000

.ak06_50 0.000 0.000 0.000

.ak06_51 0.000 0.000 0.000

.ak06_52 0.000 0.000 0.000

.ak06_53 0.000 0.000 0.000

.ak06_54 0.000 0.000 0.000

.ak06_56 0.000 0.000 0.000

.ak06_57 0.000 0.000 0.000

.ak06_58 0.000 0.000 0.000

.ak06_59 0.000 0.000 0.000

.ak06_60 0.000 0.000 0.000

.ak06_62 0.000 0.000 0.000

.ak06_63 0.000 0.000 0.000

.ak06_64 0.000 0.000 0.000

.ak06_65 0.000 0.000 0.000

.ak06_66 0.000 0.000 0.000

.ak06_67 0.000 0.000 0.000

.ak06_68 0.000 0.000 0.000

.ak06_69 0.000 0.000 0.000

.ak06_70 0.000 0.000 0.000

.ak06_71 0.000 0.000 0.000

.ak06_72 0.000 0.000 0.000

.ak06_73 0.000 0.000 0.000

.ak06_74 0.000 0.000 0.000

.ak06_75 0.000 0.000 0.000

.ak06_76 0.000 0.000 0.000

.ak06_77 0.000 0.000 0.000

.ak06_78 0.000 0.000 0.000

.ak06_79 0.000 0.000 0.000

.ak06_80 0.000 0.000 0.000

.ak06_81 0.000 0.000 0.000

.ak06_82 0.000 0.000 0.000

.ak06_83 0.000 0.000 0.000

.ak06_84 0.000 0.000 0.000

.ak06_85 0.000 0.000 0.000

.ak06_86 0.000 0.000 0.000

.ak06_87 0.000 0.000 0.000

.ak06_88 0.000 0.000 0.000

.ak06_89 0.000 0.000 0.000

.ak06_90 0.000 0.000 0.000

.ak06_91 0.000 0.000 0.000

.ak06_92 0.000 0.000 0.000

.ak06_93 0.000 0.000 0.000

.ak06_94 0.000 0.000 0.000

.ak06_95 0.000 0.000 0.000

.ak06_96 0.000 0.000 0.000

.ak06_97 0.000 0.000 0.000

.ak06_98 0.000 0.000 0.000

.ak06_99 0.000 0.000 0.000

.de02_01 0.000 0.000 0.000

.de02_02 0.000 0.000 0.000

.de02_03 0.000 0.000 0.000

.de02_04 0.000 0.000 0.000

.de02_05 0.000 0.000 0.000

.de02_06 0.000 0.000 0.000

.de02_07 0.000 0.000 0.000

.de02_08 0.000 0.000 0.000

.de02_09 0.000 0.000 0.000

.an01_01 0.000 0.000 0.000

.an01_02 0.000 0.000 0.000

.an01_03 0.000 0.000 0.000

.an01_04 0.000 0.000 0.000

.an01_05 0.000 0.000 0.000

.an01_06 0.000 0.000 0.000

.an01_07 0.000 0.000 0.000

Activity 0.000 0.000 0.000

.Depression 0.000 0.000 0.000

.Anxiety 0.000 0.000 0.000

Thresholds:

Estimate Std.Err z-value P(>|z|) Std.lv Std.all

ak06_01|t1 -0.203 0.030 -6.853 0.000 -0.203 -0.203

ak06_03|t1 -0.543 0.031 -17.466 0.000 -0.543 -0.543

ak06_04|t1 1.036 0.036 28.793 0.000 1.036 1.036

ak06_05|t1 0.082 0.029 2.771 0.006 0.082 0.082

ak06_06|t1 1.279 0.040 31.882 0.000 1.279 1.279

ak06_07|t1 -0.128 0.030 -4.320 0.000 -0.128 -0.128

ak06_08|t1 0.343 0.030 11.391 0.000 0.343 0.343

ak06_09|t1 0.315 0.030 10.504 0.000 0.315 0.315

ak06_10|t1 0.011 0.029 0.376 0.707 0.011 0.011

ak06_11|t1 0.963 0.035 27.520 0.000 0.963 0.963

ak06_12|t1 0.618 0.032 19.570 0.000 0.618 0.618

ak06_13|t1 0.844 0.034 25.128 0.000 0.844 0.844

ak06_14|t1 0.272 0.030 9.102 0.000 0.272 0.272

ak06_15|t1 0.328 0.030 10.925 0.000 0.328 0.328

ak06_16|t1 0.704 0.032 21.829 0.000 0.704 0.704

ak06_18|t1 0.990 0.035 28.004 0.000 0.990 0.990

ak06_19|t1 0.287 0.030 9.616 0.000 0.287 0.287

ak06_20|t1 0.489 0.031 15.899 0.000 0.489 0.489

ak06_21|t1 1.844 0.057 32.200 0.000 1.844 1.844

ak06_22|t1 0.420 0.030 13.813 0.000 0.420 0.420

ak06_23|t1 0.218 0.030 7.322 0.000 0.218 0.218

ak06_24|t1 0.891 0.034 26.109 0.000 0.891 0.891

ak06_25|t1 0.726 0.032 22.365 0.000 0.726 0.726

ak06_26|t1 0.094 0.029 3.194 0.001 0.094 0.094

ak06_27|t1 0.729 0.032 22.454 0.000 0.729 0.729

ak06_28|t1 -0.260 0.030 -8.727 0.000 -0.260 -0.260

ak06_29|t1 -0.018 0.029 -0.611 0.541 -0.018 -0.018

ak06_30|t1 0.437 0.030 14.324 0.000 0.437 0.437

ak06_31|t1 0.806 0.033 24.262 0.000 0.806 0.806

ak06_34|t1 0.392 0.030 12.929 0.000 0.392 0.392

ak06_35|t1 0.794 0.033 24.000 0.000 0.794 0.794

ak06_36|t1 0.492 0.031 15.991 0.000 0.492 0.492

ak06_37|t1 0.548 0.031 17.604 0.000 0.548 0.548

ak06_38|t1 0.817 0.033 24.523 0.000 0.817 0.817

ak06_39|t1 2.045 0.067 30.325 0.000 2.045 2.045

ak06_41|t1 -0.387 0.030 -12.790 0.000 -0.387 -0.387

ak06_42|t1 0.585 0.031 18.658 0.000 0.585 0.585

ak06_43|t1 -0.352 0.030 -11.671 0.000 -0.352 -0.352

ak06_44|t1 -0.337 0.030 -11.205 0.000 -0.337 -0.337

ak06_45|t1 0.532 0.031 17.144 0.000 0.532 0.532

ak06_46|t1 1.136 0.038 30.279 0.000 1.136 1.136

ak06_47|t1 0.588 0.031 18.749 0.000 0.588 0.588

ak06_48|t1 0.676 0.032 21.110 0.000 0.676 0.676

ak06_49|t1 0.697 0.032 21.649 0.000 0.697 0.697

ak06_50|t1 0.295 0.030 9.850 0.000 0.295 0.295

ak06_51|t1 1.447 0.044 32.953 0.000 1.447 1.447

ak06_52|t1 1.062 0.036 29.215 0.000 1.062 1.062

ak06_53|t1 0.779 0.033 23.649 0.000 0.779 0.779

ak06_54|t1 0.779 0.033 23.649 0.000 0.779 0.779

ak06_56|t1 0.719 0.032 22.186 0.000 0.719 0.719

ak06_57|t1 0.920 0.034 26.696 0.000 0.920 0.920

ak06_58|t1 0.489 0.031 15.899 0.000 0.489 0.489

ak06_59|t1 0.885 0.034 25.982 0.000 0.885 0.885

ak06_60|t1 0.577 0.031 18.429 0.000 0.577 0.577

ak06_62|t1 0.912 0.034 26.529 0.000 0.912 0.912

ak06_63|t1 0.617 0.032 19.525 0.000 0.617 0.617

ak06_64|t1 0.434 0.030 14.231 0.000 0.434 0.434

ak06_65|t1 1.179 0.038 30.829 0.000 1.179 1.179

ak06_66|t1 1.117 0.037 30.029 0.000 1.117 1.117

ak06_67|t1 0.615 0.032 19.479 0.000 0.615 0.615

ak06_68|t1 1.264 0.040 31.739 0.000 1.264 1.264

ak06_69|t1 0.253 0.030 8.493 0.000 0.253 0.253

ak06_70|t1 0.858 0.034 25.428 0.000 0.858 0.858

ak06_71|t1 0.105 0.030 3.569 0.000 0.105 0.105

ak06_72|t1 1.398 0.043 32.727 0.000 1.398 1.398

ak06_73|t1 0.360 0.030 11.951 0.000 0.360 0.360

ak06_74|t1 1.653 0.050 33.106 0.000 1.653 1.653

ak06_75|t1 0.905 0.034 26.403 0.000 0.905 0.905

ak06_76|t1 1.219 0.039 31.284 0.000 1.219 1.219

ak06_77|t1 1.236 0.039 31.470 0.000 1.236 1.236

ak06_78|t1 1.355 0.042 32.472 0.000 1.355 1.355

ak06_79|t1 -0.429 0.030 -14.092 0.000 -0.429 -0.429

ak06_80|t1 0.522 0.031 16.868 0.000 0.522 0.522

ak06_81|t1 0.325 0.030 10.831 0.000 0.325 0.325

ak06_82|t1 0.963 0.035 27.520 0.000 0.963 0.963

ak06_83|t1 0.283 0.030 9.476 0.000 0.283 0.283

ak06_84|t1 2.118 0.072 29.457 0.000 2.118 2.118

ak06_85|t1 1.884 0.059 31.902 0.000 1.884 1.884

ak06_86|t1 0.256 0.030 8.587 0.000 0.256 0.256

ak06_87|t1 1.062 0.036 29.215 0.000 1.062 1.062

ak06_88|t1 2.203 0.078 28.323 0.000 2.203 2.203

ak06_89|t1 1.060 0.036 29.177 0.000 1.060 1.060

ak06_90|t1 0.918 0.034 26.654 0.000 0.918 0.918

ak06_91|t1 0.362 0.030 11.998 0.000 0.362 0.362

ak06_92|t1 0.748 0.033 22.898 0.000 0.748 0.748

ak06_93|t1 1.587 0.048 33.192 0.000 1.587 1.587

ak06_94|t1 1.767 0.054 32.681 0.000 1.767 1.767

ak06_95|t1 1.670 0.051 33.066 0.000 1.670 1.670

ak06_96|t1 0.369 0.030 12.231 0.000 0.369 0.369

ak06_97|t1 0.142 0.030 4.790 0.000 0.142 0.142

ak06_98|t1 0.469 0.031 15.297 0.000 0.469 0.469

ak06_99|t1 1.627 0.049 33.155 0.000 1.627 1.627

de02_01|t1 -0.690 0.032 -21.470 0.000 -0.690 -0.690

de02_01|t2 0.703 0.032 21.784 0.000 0.703 0.703

de02_01|t3 1.412 0.043 32.803 0.000 1.412 1.412

de02_02|t1 -0.346 0.030 -11.485 0.000 -0.346 -0.346

de02_02|t2 0.837 0.034 24.956 0.000 0.837 0.837

de02_02|t3 1.409 0.043 32.785 0.000 1.409 1.409

de02_03|t1 -0.434 0.030 -14.231 0.000 -0.434 -0.434

de02_03|t2 0.402 0.030 13.255 0.000 0.402 0.402

de02_03|t3 1.045 0.036 28.947 0.000 1.045 1.045

de02_04|t1 -0.821 0.033 -24.610 0.000 -0.821 -0.821

de02_04|t2 0.375 0.030 12.417 0.000 0.375 0.375

de02_04|t3 1.097 0.037 29.738 0.000 1.097 1.097

de02_05|t1 -0.240 0.030 -8.072 0.000 -0.240 -0.240

de02_05|t2 0.600 0.031 19.069 0.000 0.600 0.600

de02_05|t3 1.308 0.041 32.127 0.000 1.308 1.308

de02_06|t1 0.299 0.030 9.990 0.000 0.299 0.299

de02_06|t2 1.031 0.036 28.715 0.000 1.031 1.031

de02_06|t3 1.572 0.047 33.194 0.000 1.572 1.572

de02_07|t1 -0.267 0.030 -8.961 0.000 -0.267 -0.267

de02_07|t2 0.695 0.032 21.604 0.000 0.695 0.695

de02_07|t3 1.351 0.042 32.449 0.000 1.351 1.351

de02_08|t1 0.706 0.032 21.873 0.000 0.706 0.706

de02_08|t2 1.455 0.044 32.982 0.000 1.455 1.455

de02_08|t3 2.056 0.068 30.195 0.000 2.056 2.056

de02_09|t1 1.199 0.039 31.060 0.000 1.199 1.199

de02_09|t2 1.822 0.056 32.353 0.000 1.822 1.822

de02_09|t3 2.289 0.085 27.082 0.000 2.289 2.289

an01_01|t1 -0.537 0.031 -17.282 0.000 -0.537 -0.537

an01_01|t2 0.772 0.033 23.473 0.000 0.772 0.772

an01_01|t3 1.401 0.043 32.747 0.000 1.401 1.401

an01_02|t1 0.057 0.029 1.926 0.054 0.057 0.057

an01_02|t2 0.954 0.035 27.356 0.000 0.954 0.954

an01_02|t3 1.553 0.047 33.187 0.000 1.553 1.553

an01_03|t1 -0.306 0.030 -10.224 0.000 -0.306 -0.306

an01_03|t2 0.762 0.033 23.252 0.000 0.762 0.762

an01_03|t3 1.351 0.042 32.449 0.000 1.351 1.351

an01_04|t1 -0.562 0.031 -18.017 0.000 -0.562 -0.562

an01_04|t2 0.497 0.031 16.130 0.000 0.497 0.497

an01_04|t3 1.168 0.038 30.694 0.000 1.168 1.168

an01_05|t1 0.137 0.030 4.649 0.000 0.137 0.137

an01_05|t2 1.045 0.036 28.947 0.000 1.045 1.045

an01_05|t3 1.664 0.050 33.080 0.000 1.664 1.664

an01_06|t1 -0.580 0.031 -18.521 0.000 -0.580 -0.580

an01_06|t2 0.579 0.031 18.475 0.000 0.579 0.579

an01_06|t3 1.334 0.041 32.330 0.000 1.334 1.334

an01_07|t1 0.080 0.029 2.724 0.006 0.080 0.080

an01_07|t2 1.055 0.036 29.100 0.000 1.055 1.055

an01_07|t3 1.592 0.048 33.190 0.000 1.592 1.592

Variances:

Estimate Std.Err z-value P(>|z|) Std.lv Std.all

.ak06_01 0.841 0.841 0.841

.ak06_03 0.830 0.830 0.830

.ak06_04 0.821 0.821 0.821

.ak06_05 0.753 0.753 0.753

.ak06_06 0.779 0.779 0.779

.ak06_07 0.826 0.826 0.826

.ak06_08 0.782 0.782 0.782

.ak06_09 0.736 0.736 0.736

.ak06_10 0.777 0.777 0.777

.ak06_11 0.753 0.753 0.753

.ak06_12 0.807 0.807 0.807

.ak06_13 0.760 0.760 0.760

.ak06_14 0.775 0.775 0.775

.ak06_15 0.771 0.771 0.771

.ak06_16 0.660 0.660 0.660

.ak06_18 0.777 0.777 0.777

.ak06_19 0.758 0.758 0.758

.ak06_20 0.651 0.651 0.651

.ak06_21 0.760 0.760 0.760

.ak06_22 0.887 0.887 0.887

.ak06_23 0.811 0.811 0.811

.ak06_24 0.746 0.746 0.746

.ak06_25 0.754 0.754 0.754

.ak06_26 0.653 0.653 0.653

.ak06_27 0.760 0.760 0.760

.ak06_28 0.754 0.754 0.754

.ak06_29 0.634 0.634 0.634

.ak06_30 0.767 0.767 0.767

.ak06_31 0.678 0.678 0.678

.ak06_34 0.682 0.682 0.682

.ak06_35 0.747 0.747 0.747

.ak06_36 0.553 0.553 0.553

.ak06_37 0.553 0.553 0.553

.ak06_38 0.750 0.750 0.750

.ak06_39 0.759 0.759 0.759

.ak06_41 0.742 0.742 0.742

.ak06_42 0.736 0.736 0.736

.ak06_43 0.704 0.704 0.704

.ak06_44 0.633 0.633 0.633

.ak06_45 0.531 0.531 0.531

.ak06_46 0.625 0.625 0.625

.ak06_47 0.670 0.670 0.670

.ak06_48 0.628 0.628 0.628

.ak06_49 0.765 0.765 0.765

.ak06_50 0.683 0.683 0.683

.ak06_51 0.741 0.741 0.741

.ak06_52 0.763 0.763 0.763

.ak06_53 0.684 0.684 0.684

.ak06_54 0.599 0.599 0.599

.ak06_56 0.637 0.637 0.637

.ak06_57 0.727 0.727 0.727

.ak06_58 0.598 0.598 0.598

.ak06_59 0.710 0.710 0.710

.ak06_60 0.778 0.778 0.778

.ak06_62 0.614 0.614 0.614

.ak06_63 0.618 0.618 0.618

.ak06_64 0.668 0.668 0.668

.ak06_65 0.699 0.699 0.699

.ak06_66 0.850 0.850 0.850

.ak06_67 0.705 0.705 0.705

.ak06_68 0.852 0.852 0.852

.ak06_69 0.836 0.836 0.836

.ak06_70 0.814 0.814 0.814

.ak06_71 0.850 0.850 0.850

.ak06_72 0.669 0.669 0.669

.ak06_73 0.765 0.765 0.765

.ak06_74 0.839 0.839 0.839

.ak06_75 0.680 0.680 0.680

.ak06_76 0.940 0.940 0.940

.ak06_77 0.602 0.602 0.602

.ak06_78 0.845 0.845 0.845

.ak06_79 0.724 0.724 0.724

.ak06_80 0.715 0.715 0.715

.ak06_81 0.648 0.648 0.648

.ak06_82 0.651 0.651 0.651

.ak06_83 0.649 0.649 0.649

.ak06_84 0.603 0.603 0.603

.ak06_85 0.780 0.780 0.780

.ak06_86 0.650 0.650 0.650

.ak06_87 0.736 0.736 0.736

.ak06_88 0.671 0.671 0.671

.ak06_89 0.695 0.695 0.695

.ak06_90 0.693 0.693 0.693

.ak06_91 0.666 0.666 0.666

.ak06_92 0.777 0.777 0.777

.ak06_93 0.691 0.691 0.691

.ak06_94 0.672 0.672 0.672

.ak06_95 0.853 0.853 0.853

.ak06_96 0.809 0.809 0.809

.ak06_97 0.814 0.814 0.814

.ak06_98 0.707 0.707 0.707

.ak06_99 0.762 0.762 0.762

.de02_01 0.467 0.467 0.467

.de02_02 0.232 0.232 0.232

.de02_03 0.580 0.580 0.580

.de02_04 0.428 0.428 0.428

.de02_05 0.681 0.681 0.681

.de02_06 0.466 0.466 0.466

.de02_07 0.490 0.490 0.490

.de02_08 0.645 0.645 0.645

.de02_09 0.481 0.481 0.481

.an01_01 0.350 0.350 0.350

.an01_02 0.174 0.174 0.174

.an01_03 0.232 0.232 0.232

.an01_04 0.335 0.335 0.335

.an01_05 0.613 0.613 0.613

.an01_06 0.547 0.547 0.547

.an01_07 0.481 0.481 0.481

Activity 0.159 0.021 7.625 0.000 1.000 1.000

.Depression 0.528 0.022 24.036 0.000 0.991 0.991

.Anxiety 0.643 0.017 37.216 0.000 0.990 0.990

Scales y*:

Estimate Std.Err z-value P(>|z|) Std.lv Std.all

ak06_01 1.000 1.000 1.000

ak06_03 1.000 1.000 1.000

ak06_04 1.000 1.000 1.000

ak06_05 1.000 1.000 1.000

ak06_06 1.000 1.000 1.000

ak06_07 1.000 1.000 1.000

ak06_08 1.000 1.000 1.000

ak06_09 1.000 1.000 1.000

ak06_10 1.000 1.000 1.000

ak06_11 1.000 1.000 1.000

ak06_12 1.000 1.000 1.000

ak06_13 1.000 1.000 1.000

ak06_14 1.000 1.000 1.000

ak06_15 1.000 1.000 1.000

ak06_16 1.000 1.000 1.000

ak06_18 1.000 1.000 1.000

ak06_19 1.000 1.000 1.000

ak06_20 1.000 1.000 1.000

ak06_21 1.000 1.000 1.000

ak06_22 1.000 1.000 1.000

ak06_23 1.000 1.000 1.000

ak06_24 1.000 1.000 1.000

ak06_25 1.000 1.000 1.000

ak06_26 1.000 1.000 1.000

ak06_27 1.000 1.000 1.000

ak06_28 1.000 1.000 1.000

ak06_29 1.000 1.000 1.000

ak06_30 1.000 1.000 1.000

ak06_31 1.000 1.000 1.000

ak06_34 1.000 1.000 1.000

ak06_35 1.000 1.000 1.000

ak06_36 1.000 1.000 1.000

ak06_37 1.000 1.000 1.000

ak06_38 1.000 1.000 1.000

ak06_39 1.000 1.000 1.000

ak06_41 1.000 1.000 1.000

ak06_42 1.000 1.000 1.000

ak06_43 1.000 1.000 1.000

ak06_44 1.000 1.000 1.000

ak06_45 1.000 1.000 1.000

ak06_46 1.000 1.000 1.000

ak06_47 1.000 1.000 1.000

ak06_48 1.000 1.000 1.000

ak06_49 1.000 1.000 1.000

ak06_50 1.000 1.000 1.000

ak06_51 1.000 1.000 1.000

ak06_52 1.000 1.000 1.000

ak06_53 1.000 1.000 1.000

ak06_54 1.000 1.000 1.000

ak06_56 1.000 1.000 1.000

ak06_57 1.000 1.000 1.000

ak06_58 1.000 1.000 1.000

ak06_59 1.000 1.000 1.000

ak06_60 1.000 1.000 1.000

ak06_62 1.000 1.000 1.000

ak06_63 1.000 1.000 1.000

ak06_64 1.000 1.000 1.000

ak06_65 1.000 1.000 1.000

ak06_66 1.000 1.000 1.000

ak06_67 1.000 1.000 1.000

ak06_68 1.000 1.000 1.000

ak06_69 1.000 1.000 1.000

ak06_70 1.000 1.000 1.000

ak06_71 1.000 1.000 1.000

ak06_72 1.000 1.000 1.000

ak06_73 1.000 1.000 1.000

ak06_74 1.000 1.000 1.000

ak06_75 1.000 1.000 1.000

ak06_76 1.000 1.000 1.000

ak06_77 1.000 1.000 1.000

ak06_78 1.000 1.000 1.000

ak06_79 1.000 1.000 1.000

ak06_80 1.000 1.000 1.000

ak06_81 1.000 1.000 1.000

ak06_82 1.000 1.000 1.000

ak06_83 1.000 1.000 1.000

ak06_84 1.000 1.000 1.000

ak06_85 1.000 1.000 1.000

ak06_86 1.000 1.000 1.000

ak06_87 1.000 1.000 1.000

ak06_88 1.000 1.000 1.000

ak06_89 1.000 1.000 1.000

ak06_90 1.000 1.000 1.000

ak06_91 1.000 1.000 1.000

ak06_92 1.000 1.000 1.000

ak06_93 1.000 1.000 1.000

ak06_94 1.000 1.000 1.000

ak06_95 1.000 1.000 1.000

ak06_96 1.000 1.000 1.000

ak06_97 1.000 1.000 1.000

ak06_98 1.000 1.000 1.000

ak06_99 1.000 1.000 1.000

de02_01 1.000 1.000 1.000

de02_02 1.000 1.000 1.000

de02_03 1.000 1.000 1.000

de02_04 1.000 1.000 1.000

de02_05 1.000 1.000 1.000

de02_06 1.000 1.000 1.000

de02_07 1.000 1.000 1.000

de02_08 1.000 1.000 1.000

de02_09 1.000 1.000 1.000

an01_01 1.000 1.000 1.000

an01_02 1.000 1.000 1.000

an01_03 1.000 1.000 1.000

an01_04 1.000 1.000 1.000

an01_05 1.000 1.000 1.000

an01_06 1.000 1.000 1.000

an01_07 1.000 1.000 1.000

Crosss-Validation

> library(haven)

> data <- read_sav("C:/Users/herzberg/Me/Artikel/Hoyer/2021/Sub_1.sav")

> View(data)

>

> model2 <- " Activity =~ ak06_01 + ak06_03 + ak06_04 + ak06_05 + ak06_06 + ak06_07 + ak06_08 + ak06_09 + ak06_10 + ak06_11 + ak06_12 + ak06_13 + ak06_14 + ak06_15 + ak06_16 + ak06_18 + ak06_19 + ak06_20 + ak06_21 + ak06_22 + ak06_23 + ak06_24 + ak06_25 + ak06_26 + ak06_27 + ak06_28 + ak06_29 + ak06_30 + ak06_31 + ak06_34 + ak06_35 + ak06_36 + ak06_37 + ak06_38 + ak06_39 + ak06_41 + ak06_42 + ak06_43 + ak06_44 + ak06_45 + ak06_46 + ak06_47 + ak06_48 + ak06_49 + ak06_50 + ak06_51 + ak06_52 + ak06_53 + ak06_54 + ak06_56 + ak06_57 + ak06_58 + ak06_59 + ak06_60 + ak06_62 + ak06_63 + ak06_64 + ak06_65 + ak06_66 + ak06_67 + ak06_68 + ak06_69 + ak06_70 + ak06_71 + ak06_72 + ak06_73 + ak06_74 + ak06_75 + ak06_77 + ak06_78 + ak06_79 + ak06_80 + ak06_81 + ak06_82 + ak06_83 + ak06_84 + ak06_85 + ak06_86 + ak06_87 + ak06_88 + ak06_89 + ak06_90 + ak06_91 + ak06_92 + ak06_93 + ak06_94 + ak06_95 + ak06_96 + ak06_97 + ak06_98 + ak06_99 "

> fit <- sem(model = model2, data = data, ordered = TRUE)

> summary(fit, standardized = TRUE, fit.measures = TRUE)

lavaan 0.6-8 ended normally after 165 iterations

Estimator DWLS

Optimization method NLMINB

Number of model parameters 182

Number of observations 1812

Model Test User Model:

Standard Robust

Test Statistic 13945.302 10385.994

Degrees of freedom 4004 4004

P-value (Chi-square) 0.000 0.000

Scaling correction factor 1.713

Shift parameter 2242.883

simple second-order correction

Model Test Baseline Model:

Test statistic 177564.844 47609.040

Degrees of freedom 4095 4095

P-value 0.000 0.000

Scaling correction factor 3.987

User Model versus Baseline Model:

Comparative Fit Index (CFI) 0.943 0.853

Tucker-Lewis Index (TLI) 0.941 0.850

Robust Comparative Fit Index (CFI) NA

Robust Tucker-Lewis Index (TLI) NA

Root Mean Square Error of Approximation:

RMSEA 0.037 0.030

90 Percent confidence interval - lower 0.036 0.029

90 Percent confidence interval - upper 0.038 0.030

P-value RMSEA <= 0.05 1.000 1.000

Robust RMSEA NA

90 Percent confidence interval - lower NA

90 Percent confidence interval - upper NA

Standardized Root Mean Square Residual:

SRMR 0.080 0.080

Parameter Estimates:

Standard errors Robust.sem

Information Expected

Information saturated (h1) model Unstructured

Latent Variables:

Estimate Std.Err z-value P(>|z|) Std.lv Std.all

Activity =~

ak06_01 1.000 0.399 0.399

ak06_03 1.034 0.085 12.197 0.000 0.413 0.413

ak06_04 1.062 0.114 9.287 0.000 0.424 0.424

ak06_05 1.246 0.100 12.469 0.000 0.497 0.497

ak06_06 1.185 0.120 9.841 0.000 0.473 0.473

ak06_07 1.038 0.088 11.776 0.000 0.414 0.414

ak06_08 1.176 0.098 11.988 0.000 0.470 0.470

ak06_09 1.295 0.103 12.523 0.000 0.517 0.517

ak06_10 1.186 0.096 12.348 0.000 0.474 0.474

ak06_11 1.266 0.114 11.129 0.000 0.505 0.505

ak06_12 1.107 0.103 10.734 0.000 0.442 0.442

ak06_13 1.226 0.108 11.375 0.000 0.490 0.490

ak06_14 1.184 0.098 12.064 0.000 0.473 0.473

ak06_15 1.189 0.097 12.272 0.000 0.475 0.475

ak06_16 1.461 0.112 13.013 0.000 0.583 0.583

ak06_18 1.202 0.110 10.976 0.000 0.480 0.480

ak06_19 1.217 0.095 12.833 0.000 0.486 0.486

ak06_20 1.485 0.109 13.643 0.000 0.593 0.593

ak06_21 1.238 0.169 7.311 0.000 0.494 0.494

ak06_22 0.843 0.089 9.432 0.000 0.337 0.337

ak06_23 1.085 0.096 11.327 0.000 0.433 0.433

ak06_24 1.267 0.109 11.631 0.000 0.506 0.506

ak06_25 1.235 0.106 11.671 0.000 0.493 0.493

ak06_26 1.462 0.106 13.812 0.000 0.584 0.584

ak06_27 1.229 0.109 11.251 0.000 0.491 0.491

ak06_28 1.232 0.102 12.096 0.000 0.492 0.492

ak06_29 1.501 0.106 14.182 0.000 0.599 0.599

ak06_30 1.212 0.099 12.265 0.000 0.484 0.484

ak06_31 1.435 0.112 12.831 0.000 0.573 0.573

ak06_34 1.419 0.104 13.623 0.000 0.567 0.567

ak06_35 1.255 0.107 11.724 0.000 0.501 0.501

ak06_36 1.659 0.116 14.288 0.000 0.663 0.663

ak06_37 1.676 0.117 14.274 0.000 0.669 0.669

ak06_38 1.254 0.110 11.387 0.000 0.501 0.501

ak06_39 1.242 0.206 6.041 0.000 0.496 0.496

ak06_41 1.262 0.097 12.979 0.000 0.504 0.504

ak06_42 1.290 0.099 12.978 0.000 0.515 0.515

ak06_43 1.356 0.101 13.434 0.000 0.542 0.542

ak06_44 1.497 0.108 13.887 0.000 0.598 0.598

ak06_45 1.709 0.121 14.125 0.000 0.683 0.683

ak06_46 1.536 0.128 12.001 0.000 0.614 0.614

ak06_47 1.441 0.108 13.324 0.000 0.576 0.576

ak06_48 1.534 0.116 13.183 0.000 0.613 0.613

ak06_49 1.211 0.108 11.265 0.000 0.484 0.484

ak06_50 1.405 0.107 13.080 0.000 0.561 0.561

ak06_51 1.289 0.135 9.544 0.000 0.515 0.515

ak06_52 1.218 0.116 10.535 0.000 0.487 0.487

ak06_53 1.425 0.112 12.683 0.000 0.569 0.569

ak06_54 1.586 0.118 13.387 0.000 0.633 0.633

ak06_56 1.521 0.112 13.570 0.000 0.607 0.607

ak06_57 1.311 0.113 11.563 0.000 0.523 0.523

ak06_58 1.583 0.115 13.714 0.000 0.632 0.632

ak06_59 1.354 0.113 12.012 0.000 0.541 0.541

ak06_60 1.170 0.096 12.124 0.000 0.467 0.467

ak06_62 1.551 0.121 12.859 0.000 0.619 0.619

ak06_63 1.556 0.115 13.576 0.000 0.621 0.621

ak06_64 1.434 0.105 13.624 0.000 0.573 0.573

ak06_65 1.373 0.125 10.972 0.000 0.548 0.548

ak06_66 0.971 0.112 8.669 0.000 0.388 0.388

ak06_67 1.361 0.110 12.412 0.000 0.543 0.543

ak06_68 0.972 0.118 8.207 0.000 0.388 0.388

ak06_69 1.010 0.094 10.694 0.000 0.403 0.403

ak06_70 1.077 0.107 10.023 0.000 0.430 0.430

ak06_71 0.961 0.092 10.399 0.000 0.384 0.384

ak06_72 1.480 0.135 10.967 0.000 0.591 0.591

ak06_73 1.211 0.099 12.194 0.000 0.484 0.484

ak06_74 1.009 0.147 6.869 0.000 0.403 0.403

ak06_75 1.421 0.110 12.934 0.000 0.568 0.568

ak06_77 1.585 0.129 12.246 0.000 0.633 0.633

ak06_78 0.982 0.121 8.082 0.000 0.392 0.392

ak06_79 1.304 0.098 13.271 0.000 0.521 0.521

ak06_80 1.329 0.109 12.196 0.000 0.531 0.531

ak06_81 1.480 0.110 13.427 0.000 0.591 0.591

ak06_82 1.484 0.122 12.211 0.000 0.593 0.593

ak06_83 1.479 0.110 13.463 0.000 0.591 0.591

ak06_84 1.571 0.226 6.941 0.000 0.627 0.627

ak06_85 1.182 0.174 6.797 0.000 0.472 0.472

ak06_86 1.483 0.104 14.215 0.000 0.592 0.592

ak06_87 1.284 0.116 11.106 0.000 0.513 0.513

ak06_88 1.441 0.223 6.452 0.000 0.576 0.576

ak06_89 1.380 0.117 11.769 0.000 0.551 0.551

ak06_90 1.386 0.117 11.805 0.000 0.553 0.553

ak06_91 1.452 0.104 13.991 0.000 0.580 0.580

ak06_92 1.188 0.103 11.486 0.000 0.474 0.474

ak06_93 1.396 0.150 9.333 0.000 0.557 0.557

ak06_94 1.442 0.170 8.490 0.000 0.576 0.576

ak06_95 0.959 0.149 6.429 0.000 0.383 0.383

ak06_96 1.092 0.089 12.250 0.000 0.436 0.436

ak06_97 1.070 0.093 11.469 0.000 0.427 0.427

ak06_98 1.355 0.107 12.706 0.000 0.541 0.541

ak06_99 1.228 0.140 8.794 0.000 0.490 0.490

> reliability(fit)

For constructs with categorical indicators, the alpha and the average variance extracted are calculated from polychoric (polyserial) correlations, not from Pearson correlations.

Activity

alpha 0.9701276

omega 0.9378468

omega2 0.9378468

omega3 0.9532352

avevar 0.2758521

| **Reliabilitätsstatistiken** | |
| --- | --- |
| Cronbachs Alpha | Anzahl der Items |
| ,935 | 91 |

| **Statistiken** | | |
| --- | --- | --- |
| ActivityM | | |
| N | Gültig | 1812 |
|  | Fehlend | 0 |
| Mittelwert | | 1,2738 |
| Median | | 1,2418 |
| Modus | | 1,20 |
| Std.-Abweichung | | ,15826 |
| Schiefe | | ,937 |
| Standardfehler der Schiefe | | ,057 |
| Kurtosis | | ,822 |
| Standardfehler der Kurtosis | | ,115 |
| Minimum | | 1,00 |
| Maximum | | 1,95 |

| **Statistiken** | | |
| --- | --- | --- |
| Activity POMP | | |
| N | Gültig | 1812 |
|  | Fehlend | 0 |
| Mittelwert | | 63,6911 |
| Median | | 62,0879 |
| Modus | | 59,89 |
| Std.-Abweichung | | 7,91289 |
| Schiefe | | ,937 |
| Standardfehler der Schiefe | | ,057 |
| Kurtosis | | ,822 |
| Standardfehler der Kurtosis | | ,115 |
| Minimum | | 50,00 |
| Maximum | | 97,25 |

| **Kolmogorov-Smirnov-Test bei einer Stichprobe** | | | |
| --- | --- | --- | --- |
|  | | | Activity Mean |
| N | | | 1812 |
| Parameter der Normalverteilung^a,b^ | Mittelwert | | 1,2738 |
|  | Std.-Abweichung | | ,15826 |
| Extremste Differenzen | Absolut | | ,095 |
|  | Positiv | | ,095 |
|  | Negativ | | -,061 |
| Teststatistik | | | ,095 |
| Asymp. Sig. (2-seitig)^c^ | | | ,000 |
| Monte-Carlo-Signifikanz (2-seitig)^d^ | Sig. | | ,000 |
|  | 99% Konfidenzintervall | Untergrenze | ,000 |
|  |  | Obergrenze | ,000 |
| a. Die zu testende Verteilung ist eine Normalverteilung. | | | |
| b. Aus den Daten berechnet. | | | |
| c. Signifikanzkorrektur nach Lilliefors. | | | |
| d. Lilliefors-Methode auf der Basis von 10000 Monte-Carlo-Stichproben mit Startwert 2000000. | | | |

Alternative estimates of reliability

Guttman bounds

L1 = 0.96

L2 = 0.97

L3 (alpha) = 0.97

L4 (max) = 0.98

L5 = 0.96

L6 (smc) = 0.99

TenBerge bounds

mu0 = 0.97 mu1 = 0.97 mu2 = 0.97 mu3 = 0.97

alpha of first PC = 0.97

estimated greatest lower bound based upon communalities= 0.99

beta found by splitHalf = 0.95

| Cronbachs Alpha | Teil 1 | Wert | ,887 |
| --- | --- | --- | --- |
|  |  | Anzahl der Items | 46^a^ |
|  | Teil 2 | Wert | ,873 |
|  |  | Anzahl der Items | 45^b^ |
|  | Gesamtzahl der Items | | 91 |
| Korrelation zwischen Formen | | | ,846 |
| Spearman-Brown-Koeffizient | Gleiche Länge | | ,917 |
|  | ungleiche Länge | | ,917 |
| Guttmans Split-Half-Koeffizient | | | ,909 |
