# Supplementary material for: Do what matters, no matter what! Factorizing positive activities during COVID-19 lockdown
Source: J Health Psychol. 2022 Sep 20;28(5):477–90. doi: 10.1177/13591053221120967 (PMC9490392; doi:10.1177/13591053221120967)
Supplement: sj-pdf-3-hpq-10.1177_13591053221120967 – Supplemental material for Do what matters, no matter what! Factorizing positive activities during COVID-19 lockdown [file sj-pdf-3-hpq-10.1177_13591053221120967.pdf]

ANTI Corona-Studie

Angenehme Tätigkeiten in der Corona-Krise

99 Aktivitäten gegen den Corona-Koller

1. Ein Bad nehmen/duschen
2. An technischen Dingen arbeiten (Auto, Fahrrad, Motorrad, Hausgeräte)
3. Einen Film/eine Serie schauen
4. Antiquitäten restaurieren, Möbel aufarbeiten
5. Ein Hörbuch/Podcast hören
6. Erzählungen, Theaterstücke oder Gedichte schreiben
7. Backen
8. Mich künstlerisch betätigen (Malen, Bildhauerei, Zeichnen)
9. Meditation oder Yoga betreiben
10. Soziale Kontakte über Social Media aufnehmen
11. Tagebuch schreiben
12. Ein Musikinstrument spielen/erlernen
13. Aktiv Pausen planen
14. Alte Fotos durchschauen/sortieren
15. Zeitschriften oder Zeitungen lesen
16. Briefe, Karten schreiben
17. Ein Lied oder ein Musikstück texten oder komponieren
18. Tipps und Ratschläge zur Selbsthilfe lesen
19. Ausgiebig Frühstücken (Brunch)
20. Sterne, Mond oder Wolken betrachten
21. Einen Zaubertrick lernen
22. Stricken, Häkeln, Sticken oder Nähen
23. Pläne für die Zeit nach Corona entwerfen
24. Mit künstlerischen Materialien arbeiten (Ton, Leder, Perlen, Wolle u.ä.)
25. Für einen guten Zweck spenden
26. Ein Vorhaben oder eine Aufgabe zu Ende bringen
27. Eine Fremdsprache lernen/üben
28. Sport treiben (z.B. Home-Workout, Gymnastik)
29. Ein neues Rezept ausprobieren
30. Puzzle spielen, Kreuzworträtsel usw. lösen
31. Ein persönliches Problem angehen
32. Videospiele spielen (z.B. Computer, Playstation, Wii)
33. Mit Haustieren spielen
34. Bewusst Musik hören (nicht nur nebenbei)
35. Einen gesunden Ernährungsplan erstellen
36. Mir Zeit für eine längere Unterhaltung nehmen
37. Jemandem Komplimente machen oder ihn loben
38. Lebensmittel einmachen, einfrieren, Vorräte anlegen
39. Etwas beichten oder für etwas Abbitte leisten
40. Garten- oder Hofarbeit verrichten
41. Hausarbeit oder Wäsche erledigen

42. Lustige Videos anschauen, Witze anhören oder lesen
43. Sauber machen und gründlich aufräumen
44. Mit Freunden oder Familienmitgliedern chatten, telefonieren oder skypen
45. Ein liebevolles Geschenk vorbereiten/machen
46. Etwas Neues lernen (z.B. jonglieren, Fußballtricks, Handstand)
47. Barfuß laufen
48. Singen
49. Meine finanziellen Angelegenheiten regeln
50. Romane, Erzählungen, Theaterstücke oder Gedichte lesen
51. Aufmerksam Radio hören
52. Landkarten studieren/Hauptstädte lernen
53. Achtsamkeitsübungen machen
54. Mir eine Aufmunterung für andere ausdenken
55. Mich intensiv (den) Kindern widmen
56. Für sich alleine tanzen
57. Fotoalbum basteln
58. Etwas ordnen oder archivieren
59. Fotografieren
60. Sexualität partnerschaftlich genießen
61. Spiele auf dem Smartphone spielen
62. Rat oder Hilfe anbieten
63. Sonnenauf- oder -untergang beobachten
64. Gesellschafts- oder Kartenspiele spielen
65. Per Internet eine "Party" mit alten Freunden organisieren
66. Fotos in sozialen Medien posten
67. Meinen Eltern eine Freude bereiten
68. Laufen, Joggen oder Freiluftübungen betreiben
69. Für eine Prüfung lernen
70. Etwas online verkaufen
71. Fahrrad fahren
72. Jemanden um Rat oder Hilfe bitten
73. Im Internet zu einem Thema recherchieren
74. Meine Homepage pflegen
75. Make-up auflegen, mein Haar richten usw.
76. Mich um neue Arbeit bewerben
77. Ein neues Hobby beginnen
78. Fernsehsendungen gründlich heraussuchen
79. Einen Spaziergang machen
80. Eine lange aufgeschobene Aufgabe umsetzen (z.B. Steuererklärung)
81. Die Wohnung verschönern oder ein Zimmer renovieren
82. Do-It-Yourself-Videos anschauen und nachmachen
83. Kleidung (aus-)sortieren oder aufwerten
84. Eine Modenschau durchführen
85. An Internet-Corona-Challenges teilnehmen (z.B. #klopapierchallenge)
86. Das Bett frisch beziehen

87. Ein Video von einem Konzert anschauen
88. Ein Gedicht oder Poetry Slam vortragen
89. Einen Online-Vortrag streamen
90. Einen Film in einer fremden Sprache anschauen
91. Mich pflegen (z.B. Gesichtsmaske, Haarkur)
92. Sexualität allein genießen
93. Ein Video drehen oder zusammenschneiden
94. Einen Karaoke-Abend (allein oder mit Familie/Mitbewohnern) machen
95. Medizinisch fasten
96. Online shoppen
97. Ein Gemüsebeet anbauen, Pflanzen umtopfen o.ä.
98. Ein Fach- oder Sachbuch lesen
99. Einen Blog-Artikel schreiben oder ein Video posten

ANTI Corona-Studie
Angenehme **T**ätigkeiten **i**n der Corona-Krise

Prof. Dr. Juergen Hoyer
Technische Universitaet Dresden
Professur für Behaviorale Psychotherapie
Institutsambulanz und Tagesklinik für Psychotherapie
Hohe Str. 53
D-01187 Dresden
