# Supplementary figures and images for: Do what matters, no matter what! Factorizing positive activities during COVID-19 lockdown
Source: J Health Psychol. 2022 Sep 20;28(5):477–90. doi: 10.1177/13591053221120967 (PMC9490392; doi:10.1177/13591053221120967)

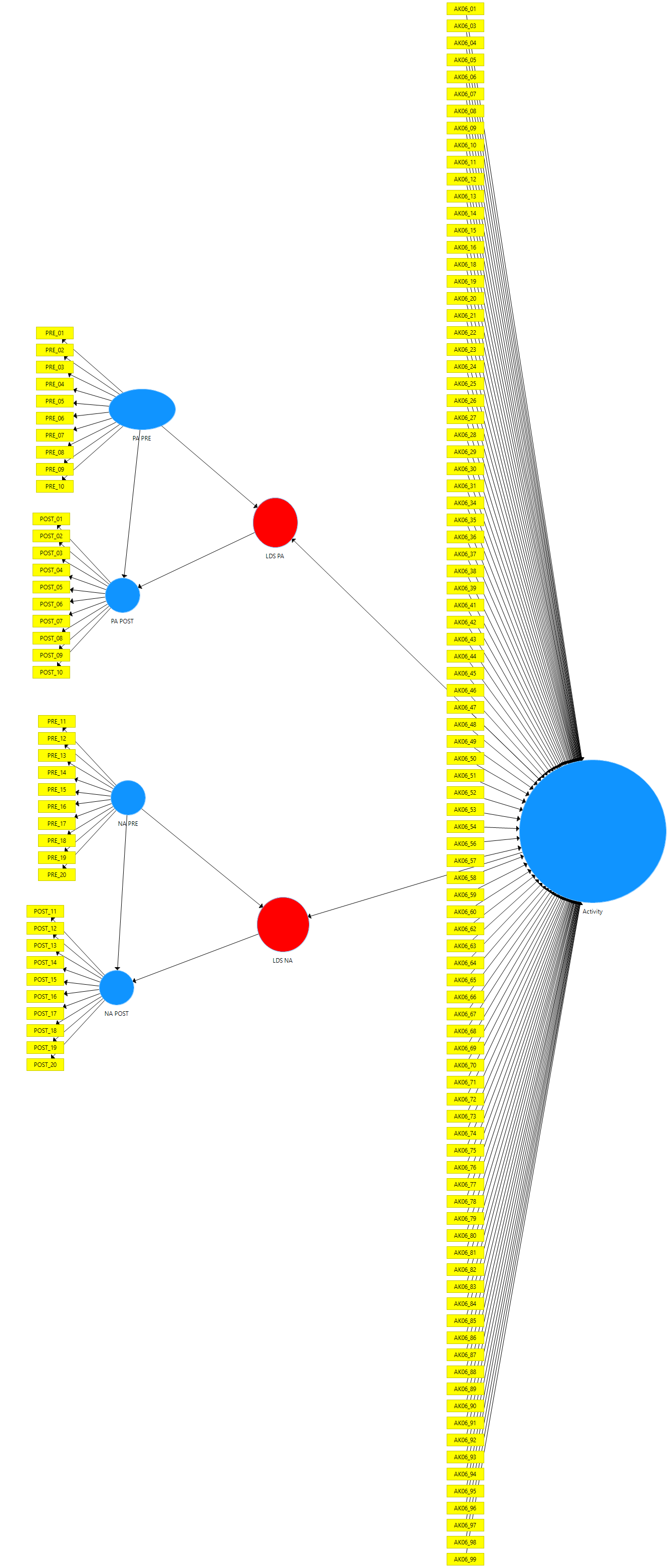

Supplement: sj-png-4-hpq-10.1177_13591053221120967 – Supplemental material for Do what matters, no matter what! Factorizing positive activities during COVID-19 lockdown [file sj-png-4-hpq-10.1177_13591053221120967.png]
